# *Prosopis cineraria* (Ghaf) Extract as a Natural Ingredient for Anti-Aging Dermocosmetic Applications

**DOI:** 10.3390/antiox15080938

**Published:** 2026-07-29

**Authors:** Beatrice De Santes, Lucia Morelli, Francesca Spena, Stefania Pagliari, Delphine Huc-Mathis, Guillaume Collet, Richard Daniellou, Marco Giustra, Luca Campone, Federico Cerri, Paolo Galli, Davide Prosperi, Lucia Salvioni, Miriam Colombo

**Affiliations:** 1 Department of Biotechnology and Bioscience, University of Milano Bicocca, 20126 Milan, Italy; b.desantes@campus.unimib.it (B.D.S.); lucia.morelli@unimib.it (L.M.); f.spena2@campus.unimib.it (F.S.); stefania.pagliari@unimib.it (S.P.); marco.giustra@unimib.it (M.G.); luca.campone@unimib.it (L.C.); davide.prosperi@unimib.it (D.P.); miriam.colombo@unimib.it (M.C.); 2 Department of Earth and Environmental Sciences, University of Milano Bicocca, 20126 Milan, Italy; federico.cerri@unimib.it (F.C.); paolo.galli@unimib.it (P.G.); 3 INRAE, AgroParisTech, UMR SayFood, Université Paris-Saclay, 91120 Palaiseau, France; delphine.huc@agroparistech.fr; 4 Cosmetology Department, AgroParisTech, 45100 Orleans, Francerichard.daniellou@agroparistech.fr (R.D.); 5 INRAE, AgroParisTech, UMR Micalis, Université Paris-Saclay, 78350 Jouy-en-Josas, France; 6 Nanobiotechnologies for Health Center, NANOMIB, University of Milano-Bicocca, 20126 Milan, Italy; 7 Marine Research and High Education (MaRHE) Center, Magoodhoo Island, Faafu Atoll 12030, Maldives

**Keywords:** *Prosopis cineraria* (Ghaf), drought- and heat-tolerant plant, antioxidant activity, anti-aging, topical cosmetic formulation

## Abstract

The ecological transition of the cosmetics industry emphasizes the need for sustainable formulations based on natural raw materials without compromising efficacy. Anti-aging skincare is a major cosmetic application, as skin aging can be accelerated by oxidative stress and extracellular matrix degradation mediated by enzymes. In this context, identifying natural ingredients with antioxidant and enzyme-modulating properties is of increasing interest. *Prosopis cineraria* (Ghaf), the national plant of the United Arab Emirates, thrives in arid environments and is hypothesized to produce bioactive secondary metabolites. Ghaf extract was evaluated for its antioxidant potential and enzyme-modulating properties relevant to skin aging. Chemical profiling (UHPLC–DAD–HRMS/MS) revealed a polyphenol-rich composition dominated by catechins and flavonoid derivatives. The extract exhibited exceptionally high radical scavenging activity in the DPPH, ABTS and ORAC assays and showed a strong correlation with its elevated total phenolic content. In addition to antioxidant effects, Ghaf extract demonstrated biologically relevant inhibition of elastase, hyaluronidase, tyrosinase and collagenase, skin-aging-related enzymes involved in extracellular matrix degradation and pigmentation. These combined in vitro activities suggest that Ghaf extract may represent a multifunctional natural ingredient for future dermocosmetic applications. Accordingly, preliminary Pickering emulsions containing the extract were formulated and characterized to demonstrate the technological feasibility of its incorporation into a topical cosmetic vehicle. These findings warrant future studies aimed at evaluating the efficacy of Ghaf-containing formulations in cellular, ex vivo and in vivo skin models.

## 1. Introduction

The cosmetics industry has experienced sustained global growth over the past decade, driven in large part by increasing consumer interest in skin-care products. This trend is closely linked not only to physical appearance but also to well-being and self-esteem, reflecting a broader societal shift that extends across genders [1]. Furthermore, market expansion has been further reinforced by demographic changes including population aging and increased life expectancy [2], which have heightened consumer demand for effective strategies to hydrate the skin, prevent premature aging, or reduce visible signs of photoaging [3].

Parallel to these demographic and economic factors, consumer awareness of sustainability, safety, and ethical production has markedly increased. Recent market analyses estimate that the global natural cosmetics sector reached approximately USD 33 billion in 2025, and natural-ingredient formulations currently account for a substantial portion of the ‘clean beauty’ segment (32.6%), reflecting a strong consumer-driven demand for sustainably sourced products [4]. As a result, there is a growing preference for cosmetic products containing plant-based, eco-friendly ingredients and manufactured using environmentally responsible extraction processes. Green extraction approaches—such as ultrasound-assisted extraction and the use of low-toxicity solvent systems—have therefore gained considerable attention [5]. Nevertheless, botanical-derived actives still represent a relatively small fraction of the global cosmetic ingredient market, underscoring the need for further research on new plant sources with potential anti-age properties.

Skin aging is a multifactorial process characterized by the gradual appearance of fine lines, wrinkles, uneven pigmentation, loss of elasticity, and dermal thinning [6]. Intrinsic factors, such as genetics and hormonal changes, contribute to these alterations; however, extrinsic factors, including ultraviolet (UV) radiation, pollution, smoking, and diet, play a predominant role in accelerating cutaneous aging, mainly by promoting the excessive production of reactive oxygen species (ROS) [7]. Oxidative stress is therefore recognized as a key driver of skin aging, representing a primary target for preventive intervention. Although the skin comes with endogenous antioxidant defense systems, these progressively decline with age and may become insufficient to contrast ROS accumulation, thus highlighting the protective value of topical supplementation with natural antioxidants [8]. Excess ROS induce oxidative damage to lipids, proteins, and DNA, while activating signaling pathways that upregulate matrix-degrading enzymes such as collagenase and elastase [9]. Degradation of collagen and elastin disrupts the structural integrity and biomechanical properties of the extracellular matrix (ECM), ultimately leading to wrinkle formation and loss of firmness [10]. Another key component of the ECM is hyaluronic acid (HA) which contributes to wrinkle reduction and skin hydration [11]. Because it is degraded by both hyaluronidase enzymes and oxidative damage, the identification of bioactive compounds capable of inhibiting hyaluronidase activity and scavenging reactive oxygen species could be crucial for preventing skin aging [12]. Furthermore, unlike collagen, HA has a relatively short half-life in the skin, meaning that treatment efficacy can be observed earlier than for collagen-targeted interventions [13]. In addition, tyrosinase is a key regulatory enzyme in the skin. By catalyzing the initial steps of melanin synthesis within melanocytes, it plays a central role in determining skin pigmentation [11]. Although melanin is essential for photoprotection, enhancing the skin’s defense against UV radiation and other environmental or hormonal factors, excessive UV exposure leads to increased ROS generation, which can further stimulate tyrosinase activity and melanin production [14]. This ROS-driven hyperactivation of melanogenesis contributes to pigmentary disorders such as lentigo, melasma, and age-related hyperpigmentation, ultimately resulting in uneven skin tone and accentuated photoaging signs [15]. Therefore, inhibitors of tyrosinase not only help prevent excessive melanin synthesis and dark spot formation, but may also indirectly attenuate oxidative stress by limiting ROS-associated melanogenic pathways. Consequently, given the central role of oxidative stress as a triggering factor for multiple aging-related cascades, there is a growing demand for cosmetic actives with strong antioxidant properties capable of counteracting ROS overproduction and, often correlated, exhibiting anti-collagenase, anti-elastase, anti-hyaluronidase, and skin-brightening activities [16].

According to these current socio-demographic trends and the growing demand for sustainable production models, plant biodiversity represents a vast and largely untapped reservoir of bioactive compounds with potential applications in skin care and anti-aging cosmetics, as capable of targeting multiple molecular pathways involved in skin aging [5]. Plants adapted to harsh environments—such as deserts, semi-arid zones, and other extreme ecosystems—often produce unique secondary metabolites that help survival under oxidative, drought, or heat stress [17]. These compounds can exhibit antioxidant [18], anti-inflammatory, and extracellular matrix-protective activities [19], making them particularly attractive for dermocosmetic formulation.

Within this context, the native flora of the United Arab Emirates (UAE) offers especially interesting opportunities. The UAE hosts a set of resilient, drought-adapted species that have evolved to withstand extreme aridity, salinity, and high temperatures [20]. Such ecological pressures often drive the accumulation of protective phytochemicals, potentially exploitable for skin care. Moreover, there is growing local interest in valorizing indigenous plants both for their ecological importance and as sustainable sources of natural ingredients [21].

Among these native species, *Prosopis cineraria*, commonly known as Ghaf, stands out as a culturally and ecologically emblematic tree. Declared the national tree of the UAE in 2008, Ghaf plays a central role in desert ecology, soil stabilization, and biodiversity preservation [22]. Traditional medicine and ethnobotanical records attribute to Ghaf a variety of therapeutic uses including anti-inflammatory, antimicrobial, and general wellness applications based on preparations derived from bark, leaves, pods, and other tissues [23]. Recent works on Ghaf’s pharmacological potential highlight its richness in flavonoids, phenolic acids, tannins, and other bioactive constituents known for antioxidant, hepatoprotective [24], and antimicrobial properties [25].

Despite this promising background, the use of Ghaf in cosmetic science remains underexplored. Few studies have addressed its phytochemical composition in detail [26], and even fewer have evaluated its potential for skin-related applications such as oxidative stress mitigation, inhibition of extracellular matrix-degrading enzymes, or formulation into stable topical systems [27].

In light of these considerations, the present study aims to explore the use of Ghaf extract as a multifunctional cosmetic ingredient. A comprehensive evaluation including (i) the characterization of its chemical profile; (ii) the assessment of antioxidant capacity and enzyme-inhibitory activity related to skin aging; and (iii) the development of a prototype topical formulation incorporating the extract, with physicochemical and stability assessment, was conducted. This work seeks to bridge ethnobotanical knowledge, desert biodiversity, and modern cosmetic science, by providing preliminary evidence that native plants like Ghaf may represent valuable sources of bioactive compounds for future dermocosmetic applications, thereby opening new research opportunities in this field.

## 2. Materials and Methods

### 2.1. Chemicals and Materials

2,2-Diphenyl-1-picrylhydrazyl (DPPH), (±)-6-Hydroxy-2,5,7,8-tetramethylchromane-2-carboxylic acid (Trolox), 2,2′-azino-bis(3-ethylbenzothiazoline-6-sulfonic acid) (ABTS), 2,2-Azobis (2-methyl-propionamidine) dihydrochloride (AAPH), fluorescein, ascorbic acid, elastase from porcine pancreas (Type IV), N-Methoxysuccinyl-Ala-Ala-Pro-Val p-nitroanilide (MAAPVN), Trizma^®^base, oleanolic acid, N,N-Dimethylformamide (DMF) anydrous, Folin & Ciocalteu’s phenol reagent, 3,4,5-Trihydroxybenzoic acid (gallic acid), sodium carbonate, collagenase from *Clostridium histolyticum* Sigma Blend Type H, N-[3-(2 Furyl)acryloyl]-Leu-Gly-Pro-Ala (FALGPA), tricine, calcium chloride dihydrate, sodium chloride, epigallocatechin gallate (ECGC), hyaluronidase type I-S, hyaluronic acid sodium salt from rooster comb, albumin from bovin serum (BSA), sodium phosphate monobasic, sodium phosphate dibasic, sodium acetate anhydrous, acetic acid, phosphate buffered saline (PBS), kojic acid, dimethyl sulfoxide (DMSO), 3,4-Dihydroxy-L-phenylalanine (L-DOPA), potassium sorbate, citric acid/sodium hydroxide/hydrogen chloride (buffer solutions for pH calibration curve), and Sodium Dodecyl Sulfate (SDS) were all purchased from Sigma-Aldrich (St. Louis, MO, USA). Ultrapure water (18 MΩ) was prepared using a Milli-Q purification system (Millipore, Bedford, MA, USA), while ethanol and methanol were purchased from CARLO ERBA Reagents s.r.l. (Milan, Italy). Acetonitrile and formic acid were purchased from Romil Chemicals (Cambridge, UK). High-glucose Dulbecco’s Modified Eagle Medium (DMEM) with and without L-glutamine, fetal bovine serum (FBS), 1% penicillin–streptomycin (P/S), L-glutamine, and 96-well and 48-well plates were purchased from EuroClone (Pero, Italy). Sunflower oil (commercial food grade Fruit d’Or) was acquired from a local retail market (Orléans, France) and used without further purification, whereas plum oil and glycerin were acquired from AromaZone (Paris, France) and VIVAPUR^®^ CS TEX EASY was kindly provided by JRS Retteinmaier (Rosenberg, Germany).

### 2.2. Plant Material

Samples of four vegetative organs of *Prosopis cineraria*, namely, twigs, roots, leaves, and bark, were collected in September 2022 from several trees of a certified nursery in Dubai (United Arab Emirates), while flower and fruits were not collected, as these tissues were not available at the time of sampling. The botanical identity of *Prosopis cineraria* was verified by Professor Massimo Labra, an expert in plant taxonomy and molecular botany. For each organ, the material collected from different trees was pooled to reduce the influence of biological variability among individual plants. Immediately after collection, all samples were freeze-dried, mechanically homogenized using a Grindomix GM 200 knife mill (Retsch, Haan, Germany), and sieved (300–600 μm; AS 200, Retsch, Hann, Germany) to obtain powders with uniform particle size distribution.

To simplify the extraction workflow and support a whole-vegetative-biomass valorization strategy, a mixed vegetative plant matrix was prepared by combining the four homogenized organ powders. The amount of each organ included in the mixture was calculated according to its relative contribution to the total freeze-dried biomass recovered from all collected vegetative organs (Table 1). Specifically, the lyophilized masses of leaves, bark, roots, and twigs were summed, and the proportion of each organ was expressed as a percentage of the total recovered biomass. These percentages were then used to prepare a 1 g composite matrix for extraction. The resulting mixed matrix was therefore intended to provide a composite representation of the selected vegetative organs included in the present study, rather than to reproduce the actual biomass distribution of the whole plant. The composite matrix was subsequently used for all extraction procedures.

### 2.3. Sample Preparation and Extraction

The Ghaf plant matrix (comprehensive of twigs, roots, leaves, and bark) thus obtained was subjected to ultrasound-assisted extraction using an ElmaSonic S 30H ultrasonic bath (Elma Schmidbauer GmbH, Singen, Germany). A 50% aqueous ethanol solution (*v*/*v*) was selected as extraction solvent due to its balanced polarity and established suitability for recovering a wide spectrum of bioactive metabolites, including phenolics (which are well known for their antioxidant properties) [28,29], while maintaining low toxicity for subsequent biological assays [30,31]. For the extraction, 1 g of plant powder was mixed with 40 mL of solvent (solid-to-solvent ratio 1:40, *w*/*v*) in a 50 mL polypropylene tube, which is commonly applied in metabolite profiling studies [32]. Ultrasonic extraction was performed at 25 °C for 15 min. To ensure exhaustive recovery of soluble compounds, the process was repeated several times with fresh solvent until colorless supernatant was obtained. After each cycle, the sample was centrifuged at 3112× *g* for 5 min using a ROTINA 380R centrifuge (Hettich Zentrifugen, Tuttlingen, Germany), and the resulting supernatant was clarified by filtration through Whatman No. 1 filter paper (pore size 7–12 µm). The combined filtrates were then concentrated under reduced pressure at 40 °C using a Hei-VAP Core rotary evaporator (Heidolph, Schwabach, Germany) to remove residual ethanol. The aqueous residue was reconstituted in Milli-Q water and subjected to a second clarification step by centrifugation at 2375× *g* for 10 min to isolate the water-soluble fraction. This strategy was adopted to obtain an aqueous extract compatible with the intended dermocosmetic formulation while minimizing residual ethanol in the final extract. The resulting supernatant was subsequently lyophilized using an Alpha 1-4 LSCplus freeze dryer (Christ, Osterode am Harz, Germany) to obtain dry extract for further characterization. Extraction yield was calculated as the percentage ratio between the weight of the dried extract and the initial dry mass of plant material.

### 2.4. Characterization of Extract

The chemical characterization of extract was performed in negative mode using a Thermo Scientific Vanquish UPLC system coupled with an Orbitrap Exploris 120 mass spectrometer (Thermo Scientific, Waltham, MA, USA). The compounds were separated using a 100 × 2.1 mm, 2.6 μm biphenyl column (Phenomenex, Torrance, CA, USA). The mobile phase consisted of 0.1% (*v*/*v*) formic acid in water (A) and 0.1% (*v*/*v*) formic acid in acetonitrile (B) at a flow rate of 0.4 mL min^−1^. The following gradient was used: 5% B (0–1 min), 5–30% B (1–7 min), 30–60% B (7–15 min), 60% B (15–18 min), 60–95% B (18–28 min) with 5 min of washing (95% B) and 5 min of equilibration (5% B) after each run. The injection volume was 5 μL for each analysis and the column was set to 30 °C. For positive ion mode, the spray voltage was set to 3400 V, while for negative ion mode it was set to −2500 V. The flow rates of the sheet gas and the auxiliary gas were set to 40 and 10 arb, respectively. The flow rate of the sweep gas was set to 2 arb. The ion transfer temperature was set to 325 °C and the vaporizer temperature to 350 °C. The full scan resolution was set to 60,000 with a scan range of m/z 100–1200 and the ddMS^2^ resolution to 45,000. The dynamic exclusion duration was set to 8 s, with an intensity threshold of 4 × 10^5^, and the collision energy was normalized to 30%. Metabolite identification followed the Metabolomics Standards Initiative (MSI) guidelines, which define three confidence levels indicated in the “IL” of HPLC-HRMS data: Level 1 (IL1): compounds were unequivocally identified by comparison with authentic reference standards (retention time, MS/MS spectrum, and exact mass); Level 2 (IL2): tentative identifications were assigned based on matches between experimental MS/MS spectra and literature data or spectral libraries (e.g., GNPS, MassBank); Level 3 (IL3): compounds were classified by spectral similarity to known chemical families and supported by taxonomic evidence; and Level 4 (IL4): unknown compounds.

### 2.5. Antioxidant Capacity and Phytochemical Content

#### 2.5.1. DPPH and ABTS Radical Scavenging Assay

The antioxidant capacity (AOC) of the Ghaf extract was quantified using the 1,1-diphenyl-2-picrylhydrazyl (DPPH^•^) radical scavenging assay, following the method described by Cerri et al. [33] with minor modifications. The extract was dissolved in ultrapure water and tested over a concentration range of 1.25–20 µg/mL, while Trolox (0–75 μM) was used as calibration standard starting from a 5 mM stock solution in methanol, subsequently diluted to generate working standards. A fresh 150 µM DPPH^•^ solution was prepared in ethanol immediately before each experiment and protected from light due to the reagent’s photosensitivity. All reactions were carried out in 96-well plates. For each measurement, 10 µL of sample, Trolox standard, or ultrapure water (blank) were added to 190 µL of the operative DPPH solution, reaching a final reaction volume of 200 µL. Plates were then incubated for 30 min at room temperature, under gentle orbital shaking (70 rpm) and protected from light. After incubation, absorbance was recorded at 517 nm using an EnSight Multimode Microplate Reader (PerkinElmer, Waltham, MA, USA).

The 2,2′-azino-bis(3-ethylbenzothiazoline-6-sulfonic acid) (ABTS) assay was conducted following the method described by Cerri et al. [33] with minor modifications. The extract was dissolved in ultrapure water and tested over a concentration range of 0.05–25 µg/mL, while Trolox (0–25 μM) was used as calibration standard starting from a 5 mM stock solution in methanol, subsequently diluted to generate working standards. A 9500 μL aliquot of 0.1 mM ABTS solution was mixed with 50 μL PBS and extract. A total of 200 μL of each mixture was pipetted into a 96-well microplate and placed in the dark for 30 min. Absorbance readings were taken at 734 nm with the microplate reader.

For both assays, the scavenging activity (%) was calculated after blank correction using the following equation:

(1) $$Scavengig\;activity\;\%=\left(1-\frac{A_{sample}}{A_{0}}\right)*100$$

where *A*_0_ corresponds to the absorbance of the zero-Trolox standard (0 µM), and *A_sample_* corresponds to the absorbance of the tested sample or Trolox concentration. A Trolox calibration curve was constructed by plotting the abovementioned activity % versus Trolox concentration (µM), yielding the equations:

(2) $$Y\;(DPPH)=0.979x+3.0218\;(R^{2}-0.9791)\;Y\;(ABTS)=0.0011x+0.007\;(R^{2}-0.9967)$$

The antioxidant activity of the Ghaf extract was then converted into Trolox Equivalent Antioxidant Capacity (TEAC) by interpolating the scavenging activity of the sample on the Trolox standard curve according to the following equation:

(3) $$TEAC\;(µM\;TX)=\left(\frac{{DPPH\;scavenging\;activy\;\%}_{sample}-intercept}{slope}\right)$$

where *slope* and *intercept* correspond to the linear regression parameter of the Trolox calibration curve. To normalize the antioxidant activity to the amount of tested material, TEAC values were further expressed as µmol Trolox equivalents per gram of dry matrix (µmol TE/g DW) by dividing the interpolated TEAC values by the corresponding extract concentration tested (g/L). This normalization allows direct comparison of antioxidant capacity independently of the tested concentration.

#### 2.5.2. Oxygen Radical Absorbance Capacity (ORAC Assay)

The ORAC assay was conducted in black 96-well plates following the method described by Cerri et al. [33] with minor modifications. Each well received 100 µL of Ghaf extract from 4 to 40 µg/mL, 100 µL of 590 mM 2,2-Azobis (2-methyl-propionamidine) dihydrochloride (AAPH) in PBS (pH 7.5), 25 µL fluorescein, and 100 µL PBS. Fluorescence (excitation at 485 nm and emission at 530 nm) was recorded every 5 min over 1 h at 37 °C. Ascorbic acid (AA) served as the reference standard. IC_50_ values (µg/mL) were based on technical triplicate measurement.

#### 2.5.3. Total Phenolic Content (TPC)

The total phenolic content of the Ghaf extract was quantified using the Folin–Ciocalteu method described by Dewanto et al. [34], 48-well microplate-adapted, with minor modifications. The extract was solubilized in 80% methanol with sonication, and 62.5 µL of the resulting solution was added to each well, together with 250 µL of ultrapure water, yielding final extract concentrations ranging from 7.8 to 62.5 µg/mL. Wells containing water instead of extract served as negative controls. Subsequently, 62.5 µL of Folin–Ciocalteu reagent (2 N) was added, and the mixture was incubated for 5 min at room temperature. Then, 62.5 µL of 7% (*w*/*v*) sodium carbonate was added to initiate color development. Plates were incubated 90 min at room temperature in the dark, after which absorbance was measured at 760 nm using a microplate reader. A standard calibration curve was generated using gallic acid dissolved in 80% methanol at final concentrations of 0.24, 0.49, 0.98, 1.95, 3.91, 7.81, and 15.63 µg/mL, yielding the equation:

(4) $$Y=0.1071x+0.0991\;(R^{2}-0.9998)$$

Total phenol content was expressed as micrograms of gallic acid equivalents per milligram of extract (μg GAE/mg). Values are expressed as mean ± standard deviation of two independent experiments, each in technical triplicate. The assay relies on the reduction of the Folin–Ciocalteu reagent by phenolic compounds under alkaline conditions, producing a blue molybdenum–tungsten complex quantifiable spectrophotometrically.

### 2.6. In Vitro Enzymatic Inhibition Assays

#### 2.6.1. Elastase Inhibition Assay

The inhibitory activity of the Ghaf extract against elastase (a serine protease involved in the degradation of elastin, a key component of the extracellular matrix) was evaluated using a spectrophotometric assay adapted from Wittenauer et al. [35], with minor modifications. The method is based on the ability of pancreatic porcine elastase to hydrolyze the synthetic chromogenic substrate N-Methoxysuccinyl-Ala-Ala-Pro-Val p-nitroanilide (MAAPVN), releasing p-nitroaniline (pNA), which can be quantified by measuring absorbance at 410 nm. A stock solution of MAAPVN (25 mg/mL) was prepared in dimethylformamide (DMF) and subsequently diluted in Tris-HCl buffer (50 mM, pH 8.0) to obtain the working substrate solution (1.328 mg/mL). Porcine pancreatic elastase was prepared at a concentration of 0.375 U/mL in the same buffer.

Assays were performed in 96-well microplates by mixing 10 µL of Ghaf extract aqueous solution with 20 µL of enzyme solution and 120 µL of buffer, resulting in final extract concentrations ranging from 12.5 to 400 µg/mL. The mixture was pre-incubated for 15 min at room temperature to allow interaction between the enzyme and the extract. Subsequently, 50 µL of the working substrate solution was added to initiate the reaction. After 15 min, absorbance at 410 nm was recorded using a microplate reader.

Elastase activity (%) was calculated by comparing the absorbance of extract-treated wells with that of enzyme controls (without inhibitor). Oleanolic acid (OA) was included as a positive control.

#### 2.6.2. Collagenase Inhibition Assay

The inhibitory activity of the Ghaf extract against collagenase was assessed using a microplate-based assay adapted from Widowati et al. [36] procedures, with minor modifications. The assay quantifies the ability of the extract to inhibit *Clostridium histolyticum collagenase* (ChC), which catalyzes the cleavage of the synthetic peptide N-[3-(2-furyl)acryloyl]-Leu-Gly-Pro-Ala (FALGPA). Hydrolysis of FALGPA results in a decrease in absorbance at 335 nm, enabling spectrophotometric monitoring of residual non-cleaved substrate. In a 96-well microplate, 30 μL of Ghaf extract aqueous solutions at concentrations ranging from 0.05 to 1.08 mg/mL were mixed with 20 μL of FALGPA 2 mM, previously diluted in methanol from a 4.1971 mM stock, in 60 µL of assay buffer (50 mM tricine, 10 mM CaCl_2_, 400 mM NaCl, pH 7.5). The plate was immediately placed in the microplate reader pre-equilibrated at 37 °C, and the absorbance at 335 nm was recorded to determine the value corresponding to 0% activity (substrate in the absence of enzyme). Collagenase was then added by dispensing 10 µL of enzyme solution (1 U/mL in cold water, freshly prepared immediately before use) into each well. Following a 20 min incubation at 37 °C, the absorbance at 335 nm was measured again. Collagenase activity (%) was calculated by comparing the decrease in absorbance in extract-treated wells with that observed in enzyme-only control wells. Epigallocatechin gallate (EGCG) was used as positive control.

#### 2.6.3. Hyaluronidase Inhibition Assay

The inhibitory effect of the Ghaf extract on hyaluronidase activity was evaluated using a turbidimetric assay adapted from Tu and Tawata [37], with minor modifications. Hyaluronidase catalyzes the hydrolytic degradation of hyaluronic acid (HA), a key glycosaminoglycan responsible for maintaining skin hydration, viscoelasticity, and structural integrity. In this assay, the remaining non-degraded HA reacts with an acidic bovine serum albumin (BSA) solution to form an insoluble precipitate whose turbidity is proportional to the amount of intact HA and can be quantified at 600 nm. Ghaf aqueous extract solutions (5 µL) were added to wells of a 48-well microplate and incubated with 100 µL of hyaluronidase type I-S (15 U/mL prepared in 20 mM sodium phosphate buffer containing 77 mM NaCl and 0.01% BSA, pH 7.0). The mixture was pre-incubated at 37 °C for 10 min to test final extract concentrations ranging from 3.7 to 66 µg/mL. Subsequently, 100 µL of HA substrate solution (0.03% *w*/*v* dissolved in 300 mM sodium phosphate buffer, pH 5.35) were added to each well, followed by a 45 min incubation at 37 °C. The reaction was terminated by adding 1 mL of acid albumin reagent (0.1% BSA, 24 mM sodium acetate, 79 mM acetic acid, pH 3.75). After a further 10 min incubation at room temperature (RT), turbidity was measured at 600 nm using a spectrophotometric microplate reader. Increased absorbance reflects higher levels of remaining HA and therefore higher inhibition of hyaluronidase activity. Hyaluronidase activity (%) was calculated by comparing sample turbidity with that of the control conditions, where 0% activity corresponds to the turbidity of HA incubated in the absence of enzyme, and 100% activity corresponds to HA fully hydrolyzed by the enzyme (untreated condition). Oleanolic acid was used as positive control.

#### 2.6.4. Human Tyrosinase Inhibition Assay

Lyophilized Ghaf extract was dissolved in PBS and serially diluted to obtain concentrations ranging from 400 µg/mL to 0 µg/mL. Kojic acid (1.4 mg/mL to 0 mg/mL) was used as a positive control. Then, 20 µL of each Ghaf extract dilution (in PBS) and positive control (in DMSO) were dispensed in triplicate into a 96-well plate. The MNT-1 human melanoma cell line (highly pigmented) was lysed with PBS containing 0.1% Triton X-100. Subsequently, 90 µL of the MNT-1 lysate, diluted to 5 × 10^5^ cells/mL in PBS, was added to each well. For blank controls (no-enzyme condition), 90 µL of PBS was added instead of lysate. Finally, 100 µL of L-DOPA substrate solution (4 mM in PBS) was added to each well. Absorbance was recorded in kinetic mode every 30 min for 18 h at 478 nm (DOPAchrome formation) and 600 nm (eumelanin formation) by using a Multiskan SkyHigh Microplate Spectrophotometer (Thermo Fischer Scientific, Waltham, MA, USA). Enzyme activity was expressed as the percentage slope normalized to the untreated condition (no extract or positive control).

### 2.7. Cell Viability Assay

#### 2.7.1. Cell Lines and Culture Conditions

The immortalized human keratinocyte cell line HaCaT was purchased from AddexBio Technologies (San Diego, CA, USA), and the highly pigmented human melanoma cell line MNT-1 was kindly provided by AgroParisTech, Chaire de Cosmétologie (Orléans, France). HaCaT cells were cultured in high-glucose Dulbecco’s Modified Eagle Medium (DMEM, ECB7501, Euroclone, Pero, Italy) supplemented with 10% heat-inactivated fetal bovine serum (FBS), 1% penicillin–streptomycin (P/S), and 2 mM L-glutamine. MNT-1 cells were maintained under the same conditions using DMEM (Gibco, 42430-025, Grand Island, NY, USA), which contains L-glutamine as supplied by the manufacturer. All cell lines were grown at 37 °C in a humidified incubator with 5% CO_2_ and 95% air.

#### 2.7.2. Viability Assay

The cytotoxicity of Ghaf extract was investigated using the in vitro colorimetric MTS assay (CellTiter 96^®^ AQ_ueous_ One Solution Cell Proliferation Assay, Promega, Madison, WI, USA) on an immortalized human keratinocyte cell line (HaCaT) following the manufacturer’s protocol. This assay detects the mitochondrial cell activity, measuring the conversion of MTS by NADPH and NADH produced by dehydrogenase enzymes in soluble formazan product, whose absorbance can be recorded at 490 nm. Briefly, 5 × 10^3^ cells in 100 μL of growth medium were seeded in a 96-well microplate. After an incubation of 24 h at 37 °C in 5% CO_2_, the medium was removed and cells were treated with 9 extract concentrations (0, 25, 50, 100, 140, 200, 270, 350 and 400 µg/mL). After 48 h of incubation, the treated medium was replaced and 20 μL of MTS solution was added. After 2 h at 37 °C in 5% CO_2_, formazan product was detected by using a microplate reader at 490 nm. Cell viability was expressed as a mean percentage compared with the untreated cells, and medium with MilliQ water at the same concentration (10% *v*/*v*) as extract-treated wells was used as blank.

### 2.8. Statistical Analysis

Statistical analyses were conducted using one-way analysis of variance (ANOVA) followed by Tukey’s honest significant difference (HSD) post hoc test. Data are presented as mean values ± standard deviation (SD). All tests assumed normal distribution and the statistical significance was considered when *p* < 0.05. Statistical analyses were performed by using GraphPad Prism (version 10.4.1).

### 2.9. Preparation of Cosmetic Emulsions

#### 2.9.1. Base Emulsion (BE) Preparation

Oil-in-water (O/W) cream emulsions (semisolid formulations) were prepared using a high-shear emulsification procedure. A combination of vegetable oils (sunflower oil and plum oil) was employed as the dispersed phase (oil phase). VIVAPUR^®^ CS TEX EASY (Pickering stabilizer) was first pre-activated in water at 2000 rpm by using a Hei-TORQUE Core mechanical stirrer (Heidolf, Schwabach, Germany). After a resting period of 15 min, the aqueous phase was completed by adding glycerin as humectant and potassium sorbate as preservative. The aqueous phase was then gradually added to the oil phase, and emulsification was performed for 3 min at 10,000 rpm using the high-shear homogenizer POLYTRON^®^ PT 10-35GT (Kinematica AG, Malters, Switzerland).

The composition % (*w*/*w*) of the BE, used as the reference formulation throughout this study, is reported in Table 2.

#### 2.9.2. Ghaf Extract Formulation

A formulation study was conducted to evaluate the physicochemical stability of the selected base emulsion containing the aqueous Ghaf extract obtained from hydroalcoholic extraction (50% EtOH/water). An aqueous solution of Ghaf extract was prepared at a concentration of 2 mg/mL and used as active ingredient. The extract solution was incorporated into the aqueous phase at inclusion levels of 3%, 5%, and 10% (*w*/*w*), corresponding to final extract concentrations in the formulation of 60, 100, and 200 μg/mL, respectively (Table 3), prior to emulsification with the oil phase.

### 2.10. Cream Characterization

Each formulation was characterized in terms of organoleptic properties, phase stability, rheological behavior, droplet size distribution analysis, pH, and accelerated stability.

#### 2.10.1. Organoleptic Characteristics

The formulations were subjected to a qualitative organoleptic inspection performed by the operator as part of the preliminary physicochemical characterization. Appearance, color, and odor were visually and olfactorily assessed under standardized conditions (fixed light, location, and background) to identify any visible or perceptible changes associated with the incorporation of the Ghaf extract.

#### 2.10.2. pH Determination

The pH of each formulation was measured 24 h after preparation, allowing the emulsions to reach the physicochemical equilibrium before analysis. Measurements were performed at RT using a pH meter 1100 L (VWR, pHenomenal^®^, Leuven, Belgium) calibrated with standard buffers at pH 4.0, 7.0, and 9.0. The electrode probe was directly immersed in the emulsion. Since the aim of this preliminary formulation study was to assess the initial suitability of the freshly prepared formulations rather than their long-term stability, pH was determined only after equilibration.

#### 2.10.3. Centrifugation (Accelerated Stability) Test

Accelerated stability was assessed by transferring ~10 g of emulsion into Falcon tubes and centrifuging at 4000 rpm for 30 min at 25 °C. Phase separation or the appearance of distinct layers was evaluated visually after centrifugation.

#### 2.10.4. Rheological Behavior

Rheological properties were studied using a Discovery HR-10 rheometer (TA Instruments, New Castle, DE, USA) by placing the cream between 40 mm diameter sandblasted plates. Two types of measurements were performed at 25 °C: flow sweep analysis by setting shear rate ranging from 0.01 to 1000 s^−1^ to measure the viscosity profile and a strain sweep oscillatory test performed at a fixed frequency of 1 Hz, with strain ranging from 0.01% to 1000% to investigate linear viscoelastic region (LVR) limits. For both analyses, samples were allowed to equilibrate for 3 min prior to measurement.

#### 2.10.5. Droplet Size Distribution Analysis

Droplet size distribution was measured using a SALD-2300 laser diffraction granulometer equipped with the SALD-MS23 Flow Cell with Sampler mode (Shimadzu, Kyoto, Japan). For each test, 2 g of emulsion was dispersed in either 18 g of water or 1% (*w*/*v*) SDS solution under magnetic stirring at room temperature for ~30 min until homogeneous dispersion was achieved. The suspension was then introduced dropwise into the flow cell. Measurements were conducted using the Fraunhofer optical model, suitable for micron-sized samples with unknown refractive indices (set to 3.00–0.20i). Each sample was analyzed in triplicate, at a light intensity corresponding to an absorbance of ~0.07. The software returned normalized droplet size distributions (µm), along with mean, mode, median, standard deviation, and D10–D50–D90 percentiles.

## 3. Results

### 3.1. Plant Extraction

To obtain a phytochemically representative extract reflecting the intrinsic metabolic diversity of Ghaf, the different anatomical parts of the plant (leaves, bark, roots, and twigs) were first collected and then pooled prior to extraction. This strategy was deliberately adopted not only to capture the complementary and potentially synergistic contribution of the distinct plant compartments but also because preliminary comparative screening indicated no substantial differences in antioxidant and enzyme-modulating activities among the individual organs. Moreover, pooling the plant fractions allows streamlining the experimental workflow and supports future scale-up toward industrially relevant processes, while simultaneously ensuring a more sustainable and value-added use of the entire vegetative plant biomass rather than selectively exploiting individual tissues. From a translational perspective, the use of a whole-vegetative-biomass valorization approach is expected to contribute to a broader functional profile and enhanced robustness of the resulting phytocomplex compared to tissue-specific extracts. To cover the widest possible polarity range and thus maximize the recovery of diverse classes of metabolites, the Ghaf mixed matrix powder was extracted exclusively using a hydroalcoholic solvent system (water–ethanol). The pooled extraction yield, expressed as percentage (*w*/*w*) of lyophilized extract relative to the dry weight of the starting plant material, was 24.83% ± 0.74 (mean ± SD, n = 7), corresponding to a relative standard deviation (RSD) of 2.99%. The low RDS indicates good repeatability of the extraction procedure across the seven independent replicates and demonstrates good reproducibility of the hydroalcoholic extraction procedure under the selected experimental conditions. The resulting pooled hydroalcoholic extract—hereafter referred to as Ghaf extract—was subsequently used for all biochemical, cellular, and formulation studies.

### 3.2. Characterization of Ghaf Extract

**Figure 1:**
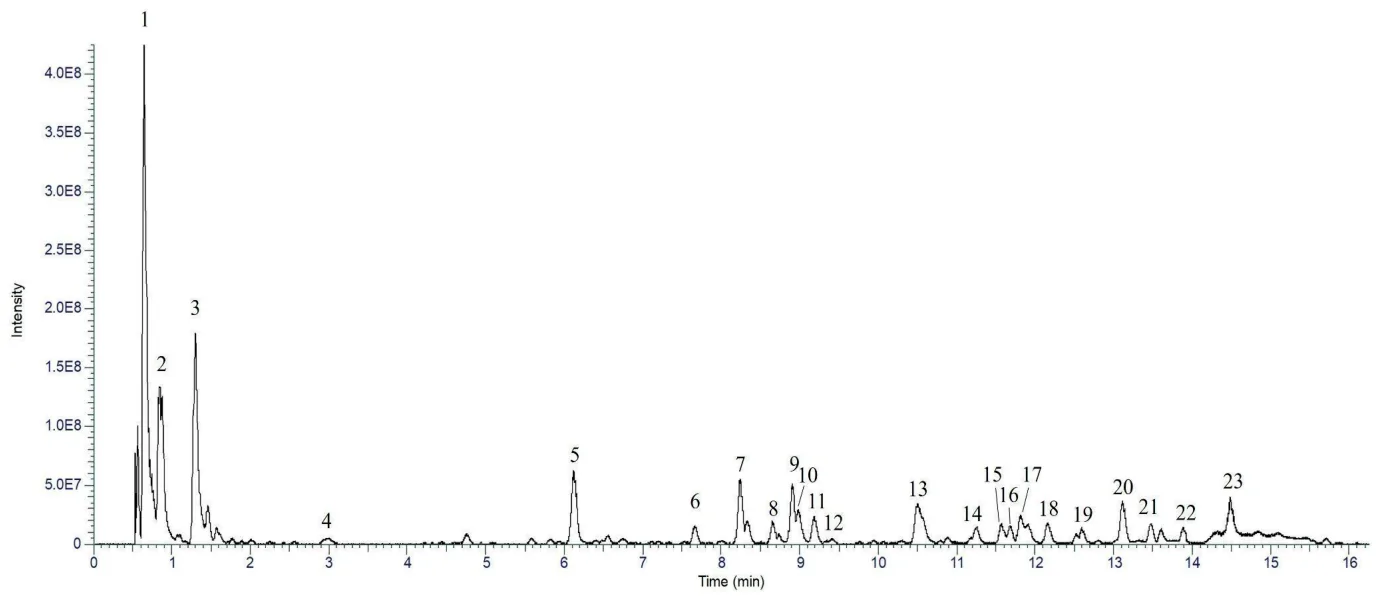
Representative chromatogram of the Ghaf extract. The intensity values are expressed using standard E notation (e.g., 1.5E8 = 1.5 × 10^8^).

The metabolite profile of Ghaf extract was analyzed by UHPLC–DAD–HRMS/MS. The chromatographic profile of the extract is reported in Figure 1 and the compounds tentatively identified are shown in Table 4, with their corresponding identification level (IL), which reflects the confidence of compound annotation based on MSI guidelines (see Section 2.4). For compounds assigned to IL2, the identification relied on comparisons of MS/MS fragmentation data with published spectra from the literature or spectral databases included directly in the table.

The analysis revealed that the Ghaf extract contains 23 compounds, which mainly belong to the phenolic compound class. Flavonoids are a heterogeneous group of polyphenols with high antioxidant activity that are widely distributed in the plant kingdom and are the most representative of the phenol compounds in the Ghaf extract. In plants, flavonoids can be either aglycones or glycosides containing hexose, deoxyhexose or pentose sugar moieties. Flavonoid glycosides can be classified as O-glycosides or C-glycosides according to their sugar linkage [50]. These can be clearly distinguished by their characteristic MS/MS fragmentation behavior in negative ion mode. C-glycosides typically exhibit neutral losses of 30, 90 and 120 Da for hexose sugars, 74 and 104 Da for deoxyhexose sugars and 60 Da for pentose sugars. Based on this diagnostic fragmentation pattern, compound 15 was tentatively identified as a C-glycoside. Specifically, the deprotonated molecular ion [M-H]^−^ at m/z 577.1574, which corresponds to the molecular formula C_27_H_30_O_14_, was assigned to 2′-O-rhamnosylvitexin. The fragment ion at m/z 457.1146 resulted from the characteristic neutral loss of 120 Da, confirming the presence of a C-linked hexose moiety. Following this fragmentation pattern the compounds 22 and 23 were identified as C-glycosides. In contrast, O-glycosides exhibit neutral losses of 162 Da (hexose sugars), 176 Da (glucuronic acid), 146 Da (deoxyhexose sugars) and 132 Da (pentose sugar). Based on this information, the compound 17 with m/z 463.0889 and molecular formula C_21_H_20_O_12_ was tentatively identified as quercetin-3-O-galactoside as indicated by the fragment ion at m/z 301.0335 which corresponded to the loss of hexose sugar (162 Da) with O-linked. Following this fragmentation pattern the compounds 13, 14, 16 and 18 were identified as O-glycosides.

A group of flavonoids is represented by flavan-3-ols, to which catechins belong. Peak 4, with an m/z of 305.0650 (C_15_H_14_O_7_) [M-H^−^], was identified as epigallocatechin, based on its fragmentation pattern [40]. The main ions produced during fragmentation were 179.0329 ([M-H-C_6_H_6_O_3_]^−^), 167.0331 ([M-H-C_7_H_6_O_3_]^−^), and 125.0230. The latter corresponds to ring A of the flavonoid. Meanwhile, peak 8 (m/z 457.0773 (C_27_H_18_O_11_)) [M-H^−^], was identified as epigallocatechin gallate, exhibiting characteristic fragments at m/z 305.0654 and 169.0128 corresponding to epigallocatechin and gallic acid, respectively.

Flavonoids and phenolic acids are known for their antioxidant activity and their ability to modulate enzymes involved in the degradation of the extracellular matrix, such as metalloproteinases (MMPs). Among the compounds present in the extract, gallic acid shows a marked inhibition of collagenase, due to the number and arrangement of hydroxyl groups on the benzene ring, which facilitate interaction with the catalytic site and chelation of the Zn^2+^ ion [10,35]. Similarly, flavan-3-ols such as catechin, epicatechin, and epicatechin gallate inhibit collagenase and elastase, with the galloyl group enhancing their activity, and also glycosidic flavonoids, such as quercetin glycosides, also modulate these proteases. Although aglycones are generally more active, glycosylation improves solubility and stability [35]. Furthermore, this class of molecules is also known for its important anti-inflammatory and immunostimulatory activity, contributing to a protective and beneficial effect [51]. Overall, the composition of the hydroalcoholic extract of Ghaf suggests the presence of bioactive molecules with anti-aging potential, justifying its selection for further functional studies.

### 3.3. Antioxidant Capacity and Phytochemical Content

#### 3.3.1. Radical Scavenging Assay

**Figure 2:**
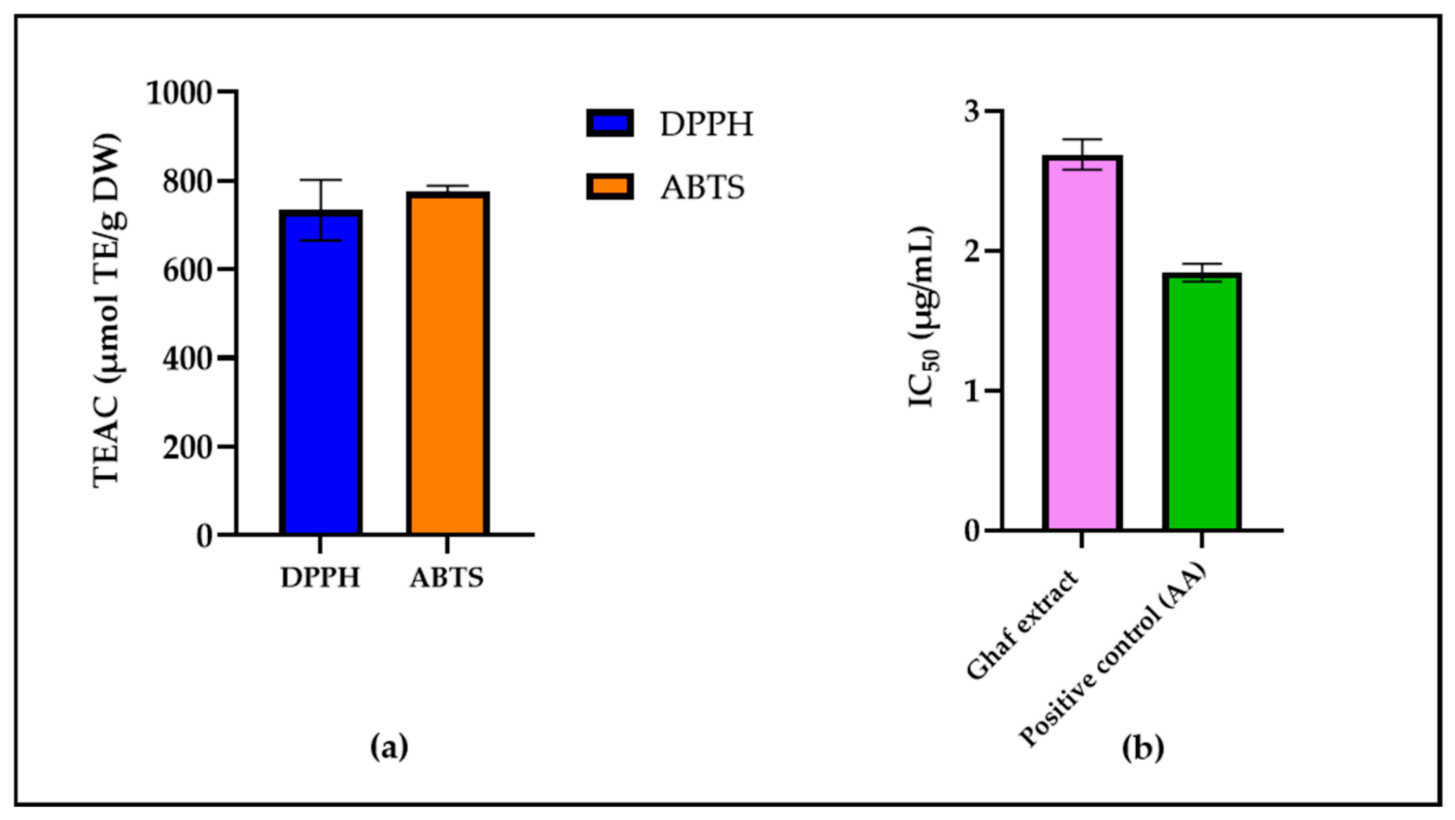
(**a**) Radical scavenging activity of the Ghaf extract expressed as Trolox equivalents (µmol TE/g DW) by using DPPH (blue) and ABTS (orange) assays. (**b**) Evaluation of antioxidant capacity of Ghaf extract by using ORAC assay, expressed as IC_50_ compared with ascorbic acid (AA) included as positive control. Values are expressed as mean ± standard deviation of two independent experiments, each in technical triplicate.

The antioxidant capacity of the Ghaf extract was assessed using three different in vitro spectrophotometric assays. The DPPH and ABTS assays were employed to evaluate the ability of the extract to neutralize different radical types in both organic and water environments, respectively. The results obtained (Figure 2a) showed high correlation between the two assays with mean values of 733.63 ± 68.04 µmol TE/g DW (DPPH) and of 775.63 ± 12.85 µmol TE/g DW (ABTS). These findings reflect a high abundance of redox-active constituents in Ghaf extract capable of donating electrons or hydrogen atoms to neutralize free radicals [52]. By comparing the results obtained by Ghaf that belongs to the *Leguminosae* with other legume species, Ghaf stands out for its high radical scavenging activity; indeed, Zhang et al. 2020 [53] reports the TEAC values from ABTS for 23 different legumes, with a range between around 8–300 µmol TE/g sample. Furthermore, the scavenging activity is notably high also in comparison with other plant extracts that exhibit TEAC values between around 0.74 and 131 TEAC (mmol/100 g DW) for ABTS assay and between around 0.39 and 164 TEAC (mmol/100 g DW) for DPPH assay [54].

To further investigate the antioxidant capacity of the Ghaf extract, another spectrophotometric assay was performed. The ORAC assay is a fluorescent kinetic method that evaluates the ability to inhibit peroxyl radical chain reaction involved in lipid peroxidation. The results in Figure 2b show the very low IC_50_ value (2.69 ± 0.11 µg/mL) of Ghaf extract. This strong inhibition of peroxyl radicals is supported by the comparison with pure ascorbic acid (IC_50_ = 1.84 ± 0.06 µg/mL) that represents one of the most antioxidant natural compounds. Considering that the extract represents a crude mixture and not a purified single compound, this antioxidant result is notable.

Collectively, this high radical scavenging capacity may be attributed to the complex phytochemical composition of the Ghaf extract, likely containing polyphenols, flavonoids acting either additively or synergistically.

#### 3.3.2. Total Phenolic Content (TPC)

Phenolic compounds, including flavonoids and phenolic acids, are known to be the major contributors to the antioxidant capacity of plants. Accordingly, the total phenolic content of Ghaf extract was also evaluated by using the Folin–Ciocalteu colorimetric assay. The total phenol content of the extract was expressed in terms of gallic acid equivalent (µg GAE/mg extract) as described in Section 2.5.3. The Ghaf extract exhibited remarkably high TPC, with a mean value obtained from two independent experiments, each in technical triplicate of 281.82 ± 14.63 µg GAE/mg of extract, indicative of a rich presence of secondary metabolites with known redox potential. Notably, preliminary extraction of the same mixed Ghaf matrix using water alone resulted in a lower total phenolic content (114.78 ± 12.26 µg GAE/mg of extract) compared to that obtained with the hydroalcoholic extraction, further supporting the selection of the hydroalcoholic solvent system for maximizing the recovery of hydrophilic phenolic constituents.

This finding aligns well with the strong antioxidant capacity previously observed in the radical scavenging assays, suggesting a close relationship between chemical composition and biological activity. Such correlation between total polyphenol content and antioxidant efficacy is consistently reported in the literature, where polyphenolic compounds (especially flavonoids, tannins, and catechins) are recognized as major contributors to the antioxidant potential of plant extracts [55].

The UHPLC–DAD–HRMS/MS analysis further confirmed the presence of abundant catechins and flavonoid derivatives, both known for their ability to donate hydrogen atoms or electrons to neutralize free radicals, as well as to chelate metal ions involved in oxidative reactions [56]. These results provide strong evidence that the antioxidant activity of the Ghaf extract can be primarily attributed to its polyphenolic fraction, although synergistic effects among different compound classes cannot be excluded.

### 3.4. In Vitro Enzymatic Inhibition Assays

The Ghaf extract was evaluated for its ability to modulate key enzymes involved in ECM degradation and melanogenesis. Elastase, collagenase, hyaluronidase, and tyrosinase were selected due to their central role in skin aging processes, including loss of elasticity, collagen breakdown, reduced hydration, and hyperpigmentation [57,58]. Since plant extracts rich in antioxidants have been shown to modulate these enzymatic pathways and display multi-target anti-aging potential [57], it was investigated whether the Ghaf extract, previously shown to possess strong antioxidant capacity, also exhibits inhibitory effects on these enzymes. Evaluating its inhibitory activity toward elastase, collagenase, hyaluronidase, and tyrosinase provides a comprehensive preliminary assessment of the extract’s in vitro biological properties relevant to skin aging. Accordingly, the multi-target potential of the extract was further investigated through the modulation of these skin-aging-related enzymes.

#### 3.4.1. Elastase Inhibition Assay

**Figure 3:**
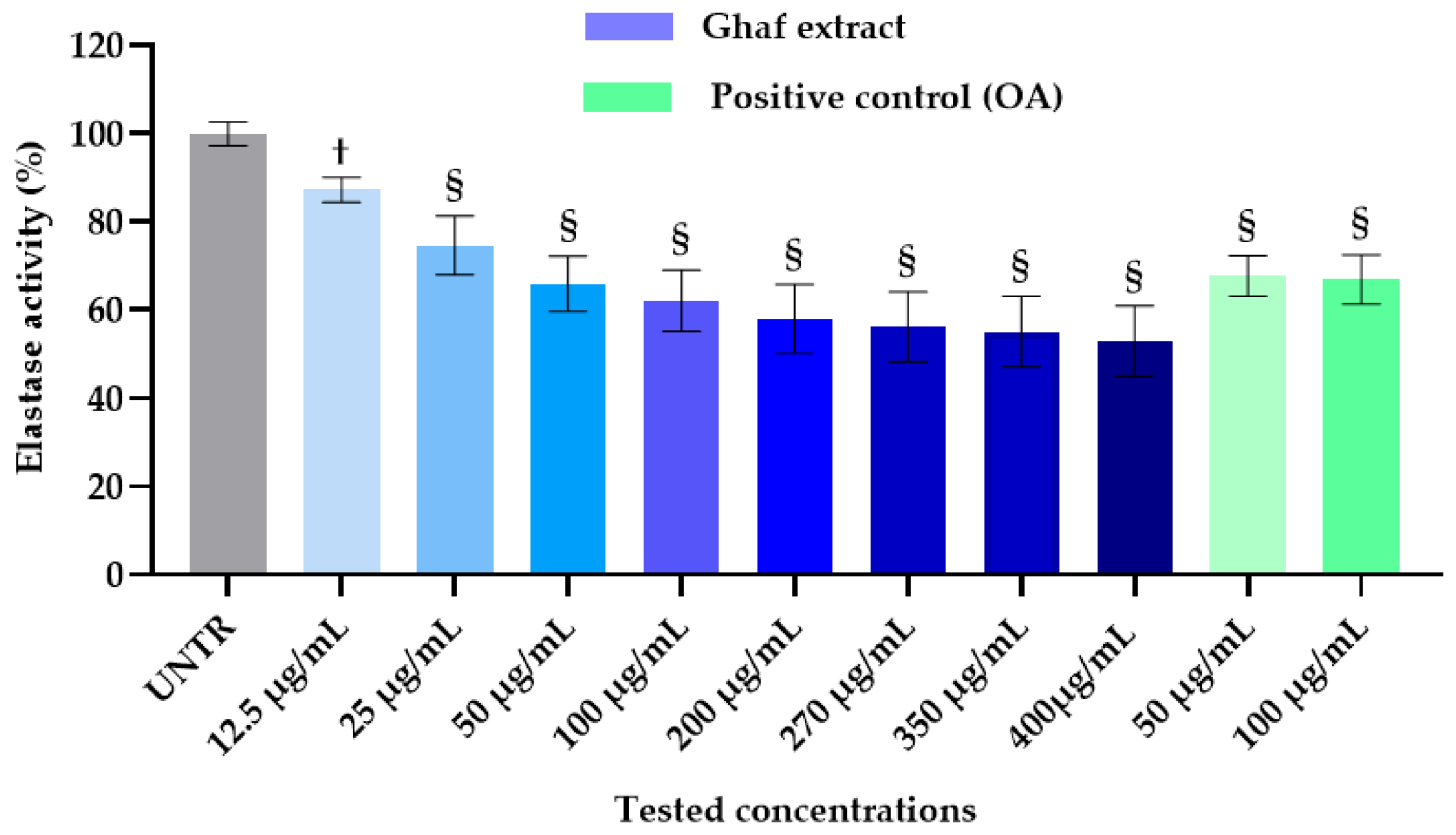
Elastase activity in presence of Ghaf extract compared to oleanolic acid (OA) used as positive control. Each bar represents the mean percentage activity ± standard deviation of two independent experiments, each in technical triplicate. Each enzyme activity reduction was significant compared to untreated condition (UNTR in grey) with *p* < 0.0001 (§), except for Ghaf at 12.5 μg/mL with *p* < 0.01 (†).

The inhibitory activity of the Ghaf extract against elastase was evaluated by quantifying enzyme activity in the presence of increasing concentrations of Ghaf extract or oleanolic acid (positive control) by measuring the amount of substrate converted by the enzyme relative to the untreated condition. A reduction in enzymatic activity therefore reflects an inhibitory effect of the tested sample. As shown in Figure 3, based on two independent experiments each performed in technical triplicate, the Ghaf extract produced a slight dose-dependent reduction in elastase activity, with inhibition becoming evident already at low concentrations (12.5–25 µg/mL) and gradually approaching a plateau around 50% residual activity at concentrations ≥ 100 µg/mL. The maximal inhibition observed was approximately 53% at the highest concentration tested (400 µg/mL). Although higher concentrations could not be tested due to solubility limitations, the inhibition trend was statistically significant (*p* < 0.05) compared to the untreated condition. Interestingly, oleanolic acid showed a comparable degree of inhibition at similar final extract concentrations, although it is a single purified compound, whereas the extract is a crude mixture. This suggests the presence of highly active constituents or synergistic interactions among phytochemicals in Ghaf extract.

A plausible explanation for the observed plateau is that a fraction of the elastase population remains catalytically active even in the presence of excess inhibitor. This behavior is typical for natural extracts, whose constituents often exhibit moderate affinity and only partially suppress enzyme activity. As a result, once the high-affinity fraction of the enzyme is inhibited, the remaining low-affinity portion continues to convert substrate, leading to a stable residual activity of approximately 50%. This phenomenon suggests that the extract achieves its maximal inhibitory effect within a relatively low concentration range, which may represent an advantage for topical applications.

#### 3.4.2. Collagenase Inhibition Assay

The collagenase inhibition activity of the Ghaf extract was also evaluated by quantifying the collagenase activity in the presence of increasing concentrations of the extract, with epigallocatechin gallate (EGCG) included as a reference inhibitor. The method relies on spectrophotometric monitoring of the decrease in absorbance of the non-cleaved synthetic substrate, where lower absorbance corresponds to greater substrate conversion by the enzyme. Enzymatic activity was therefore expressed as a percentage relative to the untreated control, allowing inhibition to be calculated accordingly.

**Figure 4:**
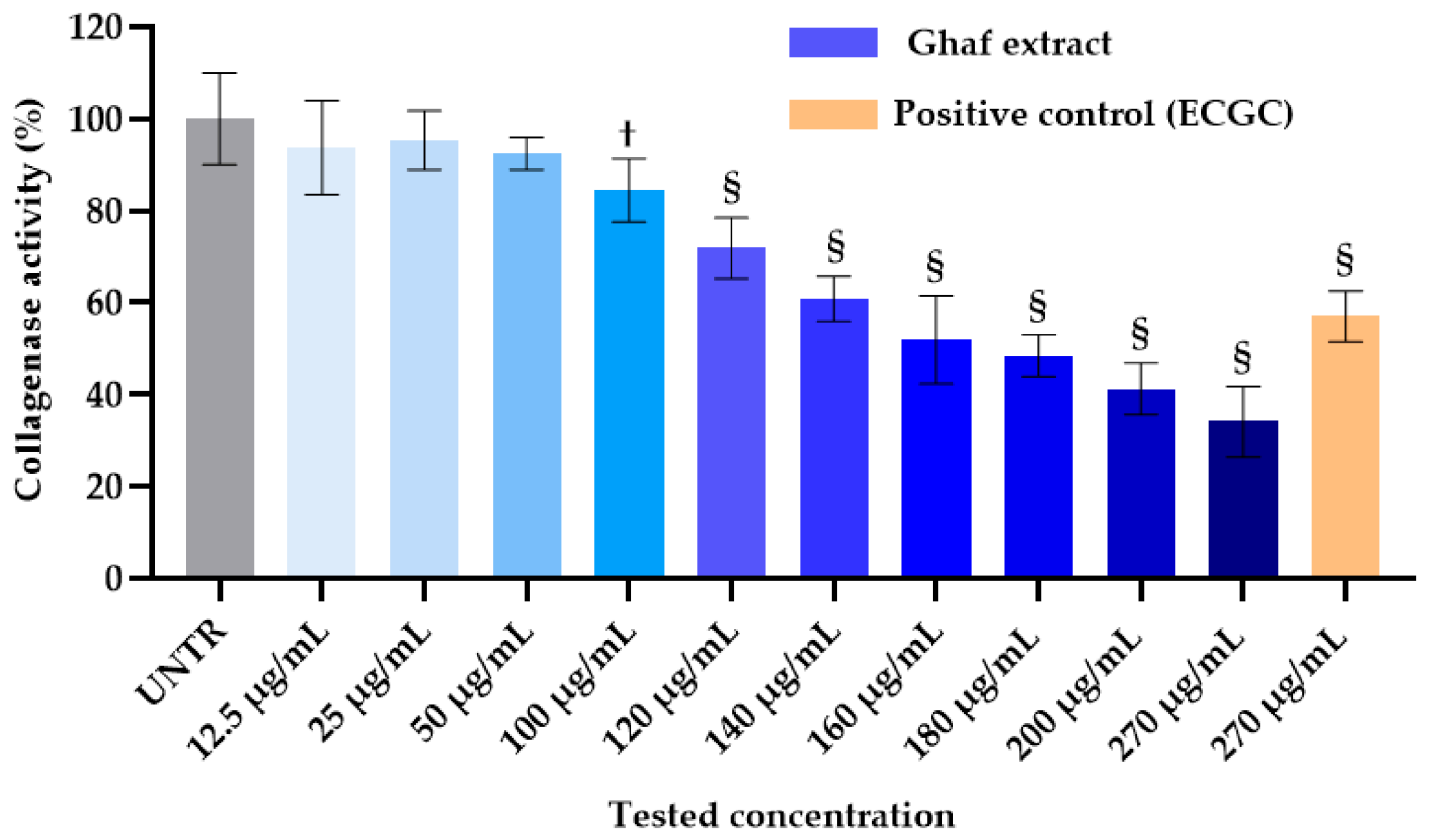
Collagenase activity in presence of increasing concentration of Ghaf extract and of epigallocatechin gallate (ECGC) as positive control. Each bar represents the mean percentage activity ± standard deviation of two independent experiments, each in technical triplicate. Each enzyme activity % was significant compared to untreated condition (UNTR in grey) with *p* < 0.0001 (§) or *p* < 0.01 (†), except for Ghaf at 12.5, 25 and 50 μg/mL.

As shown in Figure 4, data obtained from two independent experiments, each in technical triplicate, showed that Ghaf extract significantly inhibited collagenase activity starting at 100 µg/mL and displayed a clear dose-dependent trend, reaching a maximal reduction of enzymatic activity of approximately 34% at the highest tested concentration. Interestingly, EGCG exhibited lower inhibitory activity than the crude Ghaf extract at the same concentration, suggesting that the extract contains either highly potent individual constituents or synergistic phytochemical interactions that enhance its overall efficacy.

Given the key role of collagenase in the degradation of ECM proteins, such inhibition suggests that the Ghaf extract may contribute to protecting skin structural integrity and counteracting skin aging processes. Although the specific molecular components responsible for this activity remain to be identified, the results indicate the presence of constituents capable of modulating metalloproteinase activity.

#### 3.4.3. Hyaluronidase Inhibition Assay

**Figure 5:**
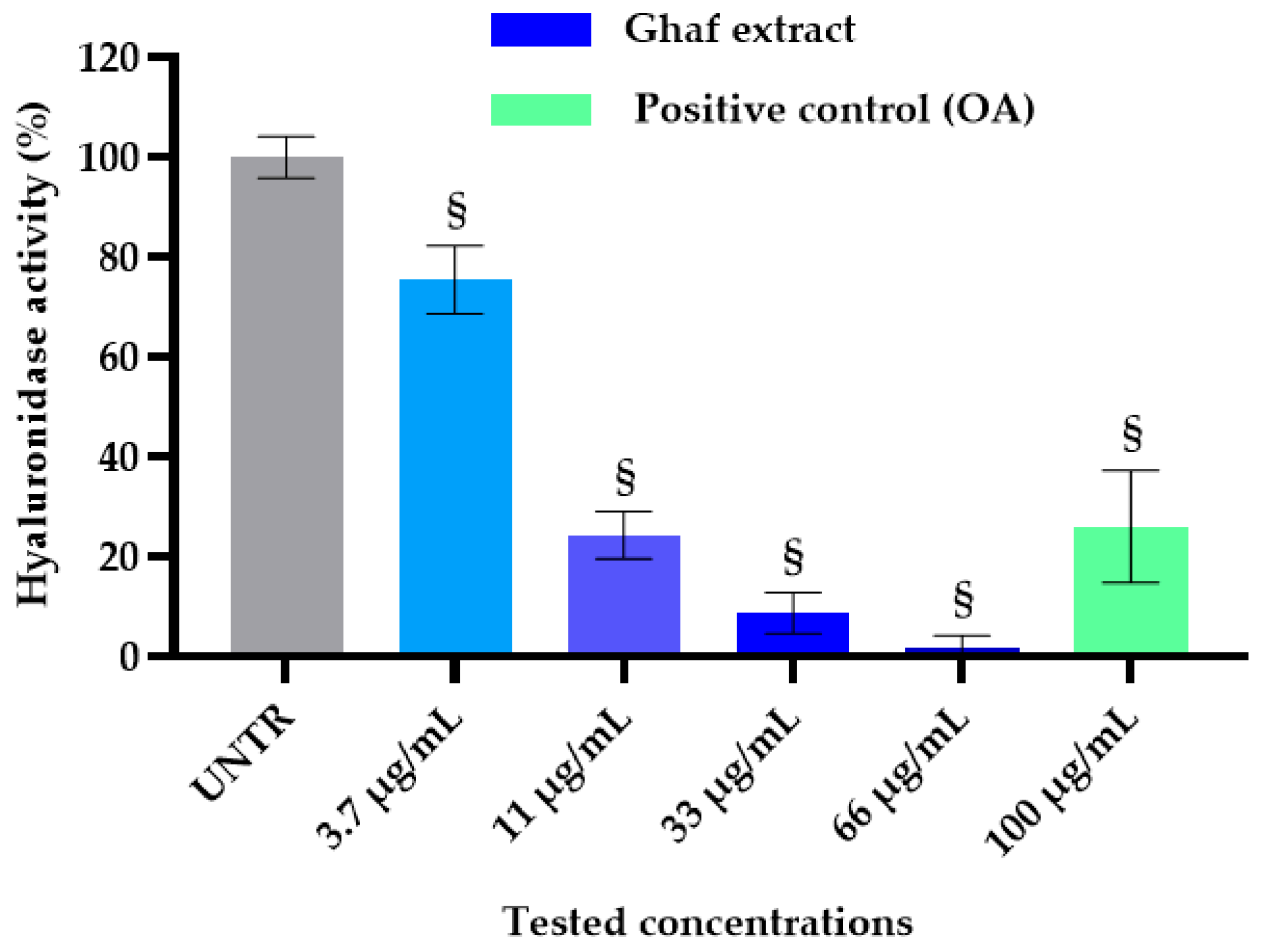
Hyaluronidase activity in presence of increasing concentration of Ghaf extract compared to oleanolic acid (OA) used as positive control. Each bar represents the mean percentage activity ± standard deviation of two independent experiments, each in technical triplicate. All enzyme activities reported were significant compared to untreated condition (UNTR in grey) with *p* < 0.0001 (§).

The hyaluronidase inhibitory activity of Ghaf extract was also evaluated. This assay quantifies the enzyme activity by measuring the turbidity generated by undigested HA in the presence of acidified BSA solution and increasing concentrations of the test inhibitor, with oleanolic acid serving as a positive control. As shown in Figure 5, results from two independent experiments performed in technical triplicate demonstrated that Ghaf extract exhibited a marked, concentration-dependent reduction of hyaluronidase activity, reaching near-complete inhibition at the highest tested concentration (66 µg/mL). Furthermore, the Ghaf extract performance was strongly higher compared to oleanolic acid, a pure compound widely used as a reference hyaluronidase inhibitor. Indeed, the inhibition observed with 100 µg/mL of oleanolic acid was comparable to that achieved with approximately 11 µg/mL of Ghaf extract. These results suggest synergistic effects among bioactive constituents. Such synergism may enhance the overall potency of the extract, making it a promising natural candidate for anti-aging and skin-protective applications.

Because hyaluronidase catalyzes the depolymerization of hyaluronic acid (a key molecule for skin hydration and viscoelasticity) [59], the strong inhibitory activity observed suggests that the extract contains constituents capable of modulating ECM degradation pathways relevant to skin aging.

#### 3.4.4. Human Tyrosinase Inhibition Assay

**Figure 6:**
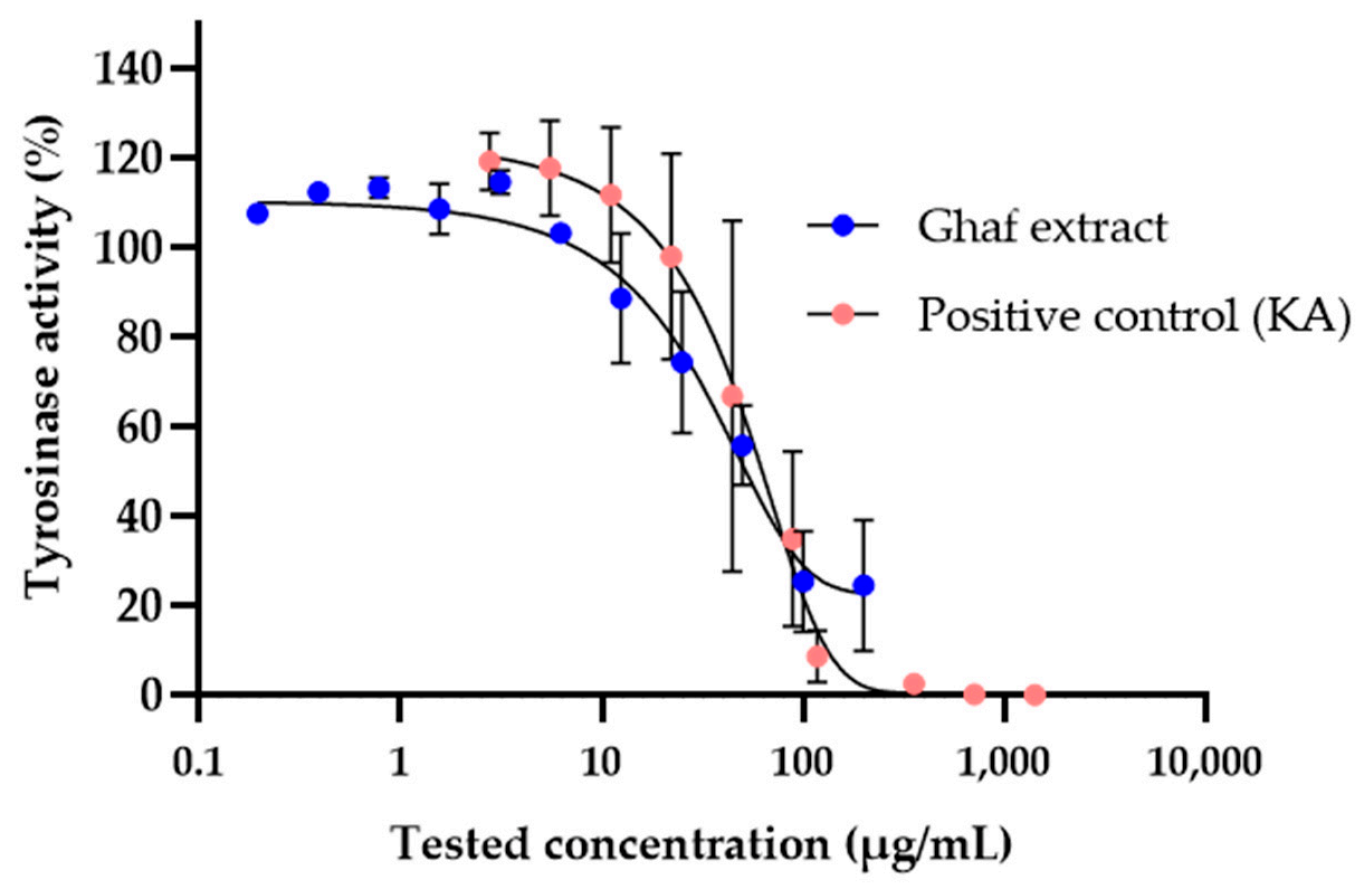
Tyrosinase inhibition by kojic acid and Ghaf extract. Dose–response inhibition curves of human tyrosinase in the presence of kojic acid (KA, positive control) and exposed to increasing concentrations of Ghaf extract. Data represent mean ± SD of three independent experiments (n = 3), each in technical triplicate.

Ghaf extract was also investigated for human cellular tyrosinase inhibiting abilities in MNT-1 cell lysate. In this assay the TYR-mediated dopachrome and eumelanin formation [60] was monitored at 478 nm and 600 nm, respectively, in the presence or absence of Ghaf extract, with kojic acid included as a positive control. The resulting typical kinetic profiles were subsequently converted into slope values normalized on the untreated condition, yielding the percentage of human tyrosinase activity. As shown in Figure 6, kojic acid produced a characteristic sigmoidal dose–response with strong inhibition, validating the sensitivity and robustness of the assay. Based on three independent experiments, each in technical triplicate, Ghaf extract similarly reduced tyrosinase activity in a concentration-dependent manner, although with lower potency, reaching an estimated IC_50_ below 100 µg/mL. Despite being less active than kojic acid, this inhibitory effect is remarkable given the crude, heterogeneous nature of the botanical extract.

Taken together, these findings suggest that the Ghaf extract contains bioactive molecules capable of modulating melanogenic enzymes, supporting its future investigation as a potential multifunctional ingredient for dermocosmetic applications, including skin-brightening strategies. Overall, the combination of the observed antioxidant and enzyme modulating activities suggest that the extract’s dermocosmetic interest may arise from complementary mechanisms.

### 3.5. Cell Viability Assay

**Figure 7:**
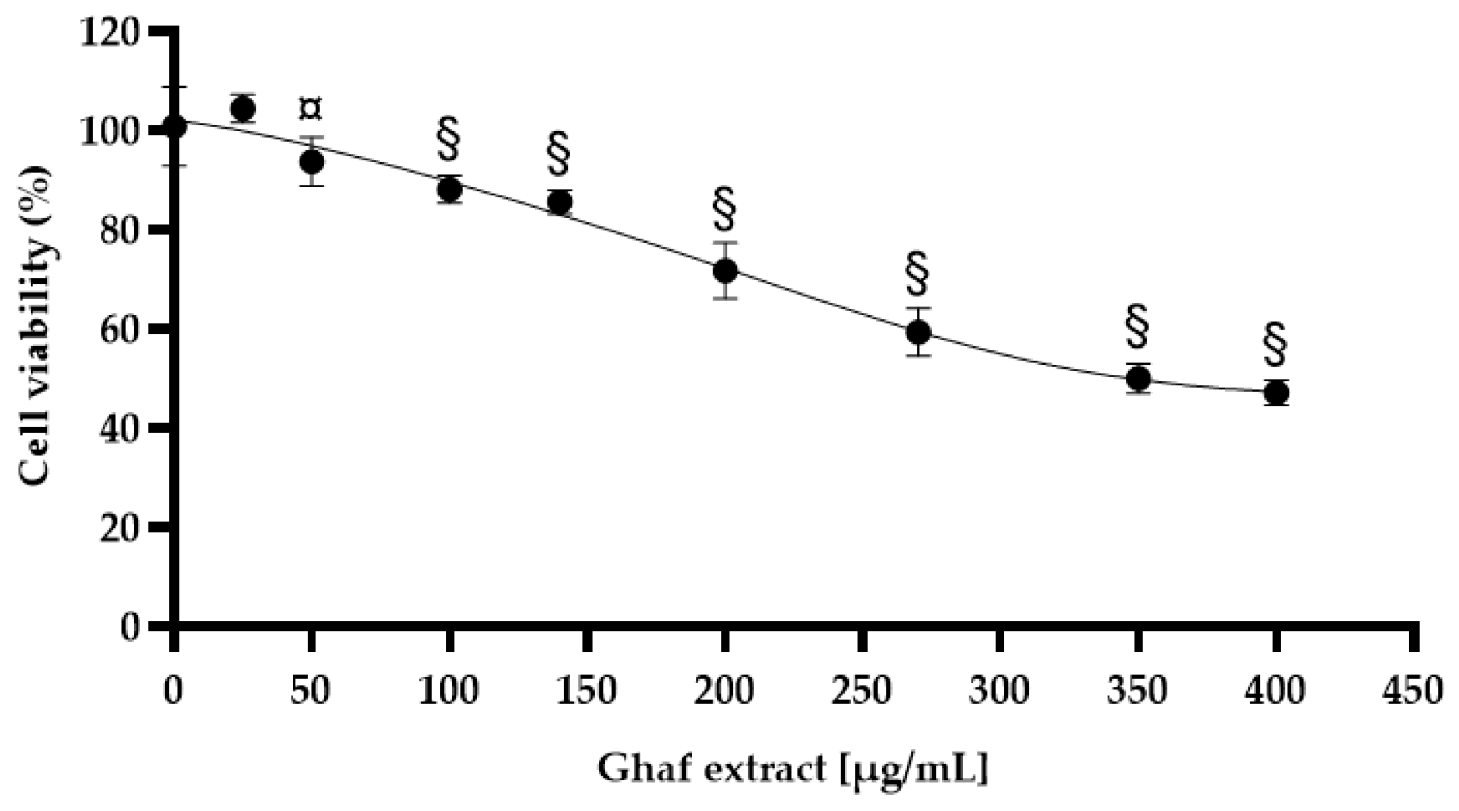
Cell viability (%) of HaCaT cell line treated with Ghaf extract (25–400 µg/mL) for 48 h compared to untreated condition. Bars represent mean percentage ± standard deviation of two independent experiments, each in technical quadruplicate. § (*p* < 0.0001) and ¤ (*p* < 0.05) vs. untreated condition.

To determine the non-cytotoxic concentration range of the Ghaf extract, HaCaT keratinocytes were treated with increasing concentrations of Ghaf extract and incubated for 48 h. Cell viability was subsequently assessed using the MTS assay, with results expressed as percentage of viable cells relative to untreated control (Figure 7).

The obtained data revealed that the extract maintained high cell viability (>70%) up to concentrations of 200 µg/mL, followed by a slow decrease reaching 47% of cell viability at the highest tested concentration. Although a cytotoxic effect is observed at higher doses, the profile suggests that the Ghaf extract does not negatively affect HaCaT cell viability within the concentration range relevant to the enzymatic and antioxidant activities observed in the aforementioned assays. These findings confirm that the extract can exert meaningful bioactivity without compromising cell viability, supporting its safe integration into topical formulations. Furthermore, cytotoxicity in monolayer cultures should be interpreted cautiously, as formulated systems typically reduce the effective bioavailable concentration of active ingredients [61,62].

### 3.6. Development and Characterization of Cosmetic Emulsions

#### 3.6.1. Base Emulsion Formulation

Based on the biochemical screening presented above which revealed the Ghaf extract’s promising in vitro antioxidant and enzyme-modulating properties relevant to skin aging, the next step of this study focused on the development of preliminary cosmetic formulations incorporating this extract, in order to evaluate the technological feasibility of its incorporation into a topical delivery system. A facial cream was selected as the delivery system, as nowadays creams together with serums represent the most widely attractive and commercially relevant cosmetic formats for facial skincare applications [63].

The first step consisted in the design of a base emulsion suitable for the incorporation of the Ghaf extract. In line with current trends in sustainable and eco-conscious skincare, the formulation was developed according to strict sustainability-driven criteria, including a minimal INCI list, avoidance of heating steps, and the exclusive use of biodegradable and naturally derived ingredients. To further enhance sustainability and skin compatibility, a Pickering emulsification strategy was adopted. This approach reduces the need for conventional synthetic surfactants and is increasingly recognized as an innovative, environmentally friendly alternative that offers improved biocompatibility, physical stability, and biodegradability [64,65]. The key stabilizing agent selected was VIVAPUR^®^ CS TEX EASY, a co-processed, natural and biodegradable powder composed of microcrystalline cellulose, xanthan gum, and cellulose. This material is capable of forming a robust interfacial stabilizing network by adsorbing at the oil–water interface, thereby efficiently stabilizing the emulsion through a particulate-based mechanism. The oil phase was intentionally kept at low levels and composed exclusively of plant-derived oils, selected for their dual role as emollients enhancing skin sensorial properties and as natural sources of fragrance.

The selected base emulsion consisted of an aqueous phase (84% *w*/*w*), containing water (74.88% *w*/*w*), glycerin (5% *w*/*w*), VIVAPUR^®^ CS TEX EASY (4% *w*/*w*), and potassium sorbate (0.2% *w*/*w*), and an oil phase (16% *w*/*w*) composed of sunflower oil (10.5% *w*/*w*) and plum oil (5.5% *w*/*w*). This formulation was selected as the reference vehicle because it fulfilled the sustainability criteria established for this study while providing satisfactory physicochemical characteristics, including phase stability, suitable pH, acceptable organoleptic properties, and appropriate rheological behavior. As this base emulsion was subsequently used for the incorporation of the Ghaf extract, its physicochemical characterization is presented together with that of Ghaf extract formulations in the following sections to facilitate direct comparison among all formulations.

#### 3.6.2. Ghaf Extract Formulation

After defining and formulating the base emulsion, the Ghaf extract was incorporated as an aqueous solution (2 mg/mL, based on its solubility in water) at three different concentrations: 3%, 5%, and 10% (*w*/*w*), corresponding to final extract concentrations in the formulation of 60, 100, and 200 μg/mL, respectively. The lowest inclusion level (3% *w*/*w*) was selected as a rational compromise between the extract concentration range commonly reported in the literature [66] for cosmetic actives and the favorable safety profile observed in preliminary cell viability assays. Given that the biological activity and tolerability of an active ingredient may change within a complex formulation matrix [61], higher extract loadings (5% and 10% *w*/*w*) were also evaluated to explore potential concentration-dependent effects.

All Ghaf-containing emulsions were subsequently characterized and compared with the base formulation in order to evaluate their physicochemical stability and organoleptic properties.

#### 3.6.3. Organoleptic Characteristics

Firstly, the organoleptic characterization of the emulsions obtained was conducted and results are summarized in Table 5. Both the base emulsion and the formulations containing the Ghaf extract retained a semi-solid, homogeneous appearance over 7 days, regardless of temperature conditions (4 °C, 25 °C, or 45 °C).

A slight color shift from the off-white of the base emulsion to a light beige/gray tone was observed at higher extract concentrations, with further intensification at elevated storage temperatures. Such behavior is consistent with natural pigment oxidation (e.g., polyphenols and flavonoids that may undergo mild oxidation or color deepening upon heat exposure) [67], but no signs of degradation or phase instability were observed.

Odor evaluation revealed an enhancement of the characteristic plum oil scent, complemented by a subtle herbal note that increased proportionally with extract content. The odor profile remained stable at all three storage temperatures, indicating the absence of extract degradation or rancidity phenomena during the observation period.

#### 3.6.4. pH Determination

At 24 h after preparation, the pH values were 5.6, 5.9, 5.8, and 5.9 for the base emulsion and for the emulsions containing 3% (*w*/*w*), 5% (*w*/*w*), and 10% (*w*/*w*) of Ghaf extract solution, respectively. Thus, all formulations remained within the dermocompatible acidic range typically recommended for topical applications [68]. Importantly, the incorporation of Ghaf extract solution therefore did not appreciably alter the pH of the base formulation, indicating that the extract does not perturb acid–base equilibria of the emulsion. This is a desirable feature in cosmetic emulsions, as it prevents irritation potential and ensures formulation stability and preservative efficacy [69].

#### 3.6.5. Centrifugation (Accelerated Stability) Test

The centrifugation test was performed on both the base emulsion and the Ghaf-containing formulations after 24 h. No phase separation or signs of creaming or sedimentation were observed in any sample. This result demonstrates that all emulsions were physically stable even under strong mechanical stress, confirming the excellent interfacial stabilization capacity of VIVAPUR^®^ CS TEX EASY. The findings suggest that the formulations possess high kinetic stability, which is a strong predictor of long-term shelf stability and robustness under real storage conditions.

#### 3.6.6. Rheological Behavior

The rheological behavior of the base emulsion and of formulations containing increasing concentrations of Ghaf extract solution at 2 mg/mL (3%, 5%, and 10% *w*/*w*) was evaluated to investigate the effect of extract incorporation on the structural integrity and viscoelastic properties of the system. Flow and oscillatory measurements were performed at 25 °C using a Discovery HR-10 rheometer, following the procedure described in Section 2.10.4.

**Figure 8:**
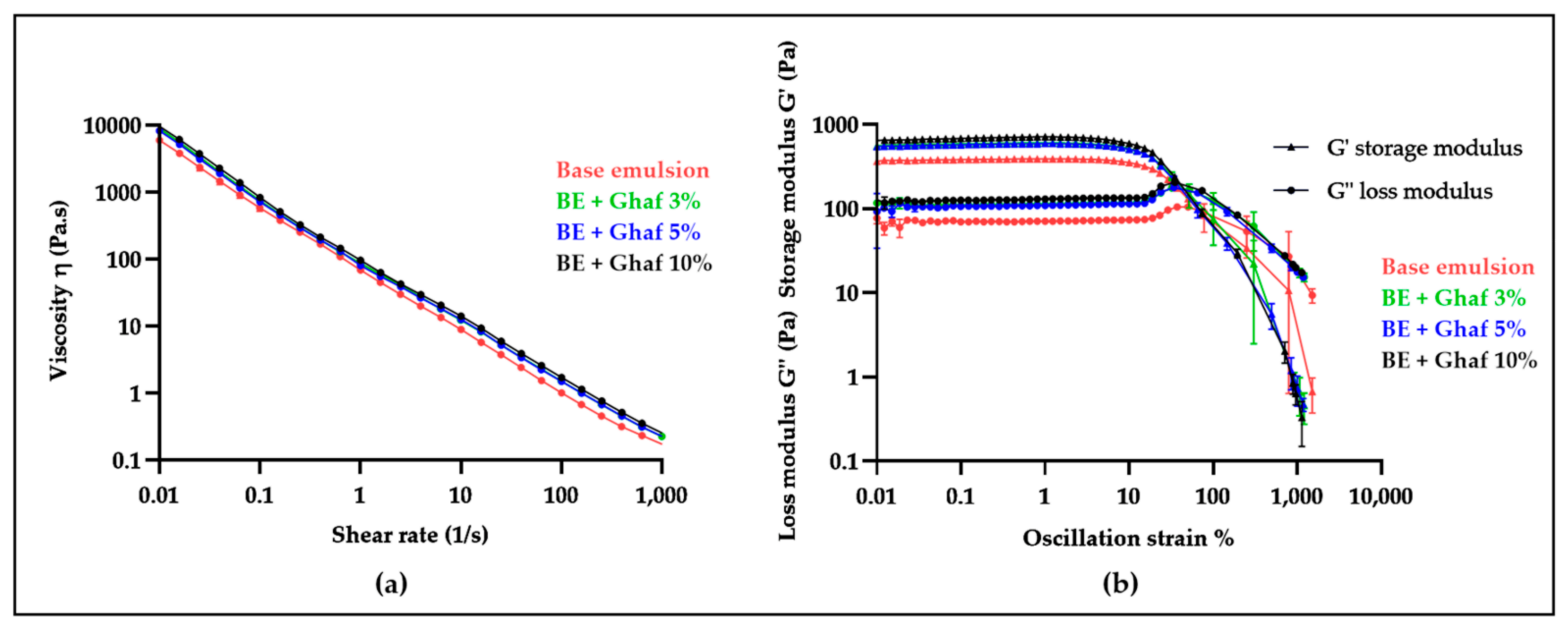
(**a**) Viscosity (η) vs. shear rate for all formulations (base emulsion in red; BE (base emulsion) with Ghaf solution at 3% (*w*/*w*) in green; with Ghaf solution at 5% (*w*/*w*) in blue; with Ghaf solution at 10% (*w*/*w*) in black). (**b**) Oscillatory strain sweep curves of storage (G′) and loss (G″) moduli as a function of oscillation strain (%). Each value represents the mean ± standard deviation of three independent measurements (n = 3).

The flow sweep analysis (Figure 8a) revealed a shear-thinning behavior for all samples, characterized by a marked decrease in viscosity with increasing shear rate—from approximately 10^4^ Pa·s at 0.01 s^−1^ down to below 1 Pa·s at 1000 s^−1^. This pseudoplastic profile is typical of Pickering-type emulsions and indicates a stable, structured network that can recover its internal organization after shear removal [70]. Importantly, the viscosity curves of the Ghaf-containing formulations perfectly overlapped with that of the base emulsion, confirming that extract solution incorporation did not significantly alter the microstructure and flow properties of the system. The consistency and slope of the viscosity profiles remained nearly identical across all tested concentrations, suggesting that the polysaccharide–cellulose interfacial network provided by VIVAPUR^®^ CS TEX EASY dominates the rheological response, even in the presence of bioactive solutes.

Oscillatory strain sweep tests (Figure 8b) further supported these findings. All formulations exhibited a linear viscoelastic region (LVR) extending up to approximately 63% strain, within which the storage modulus (G′) exceeded the loss modulus (G″), indicating predominantly elastic solid-like behavior. Beyond this point, both moduli decreased as the structure progressively broke down under increasing deformation. The near superimposition of G′ and G″ curves across all samples confirmed that the addition of the Ghaf extract solution at concentrations up to 10% (*w*/*w*) did not compromise the internal microstructure or viscoelastic balance of the emulsion.

Overall, these results demonstrate that the incorporation of Ghaf extract as aqueous solution does not negatively impact on the mechanical stability or rheological integrity of the Pickering emulsion. The formulations maintain consistent shear-thinning and viscoelastic properties, essential for good spreadability, sensory performance, and storage stability in topical cosmetic applications.

#### 3.6.7. Droplet Size Distribution Analysis

**Figure 9:**
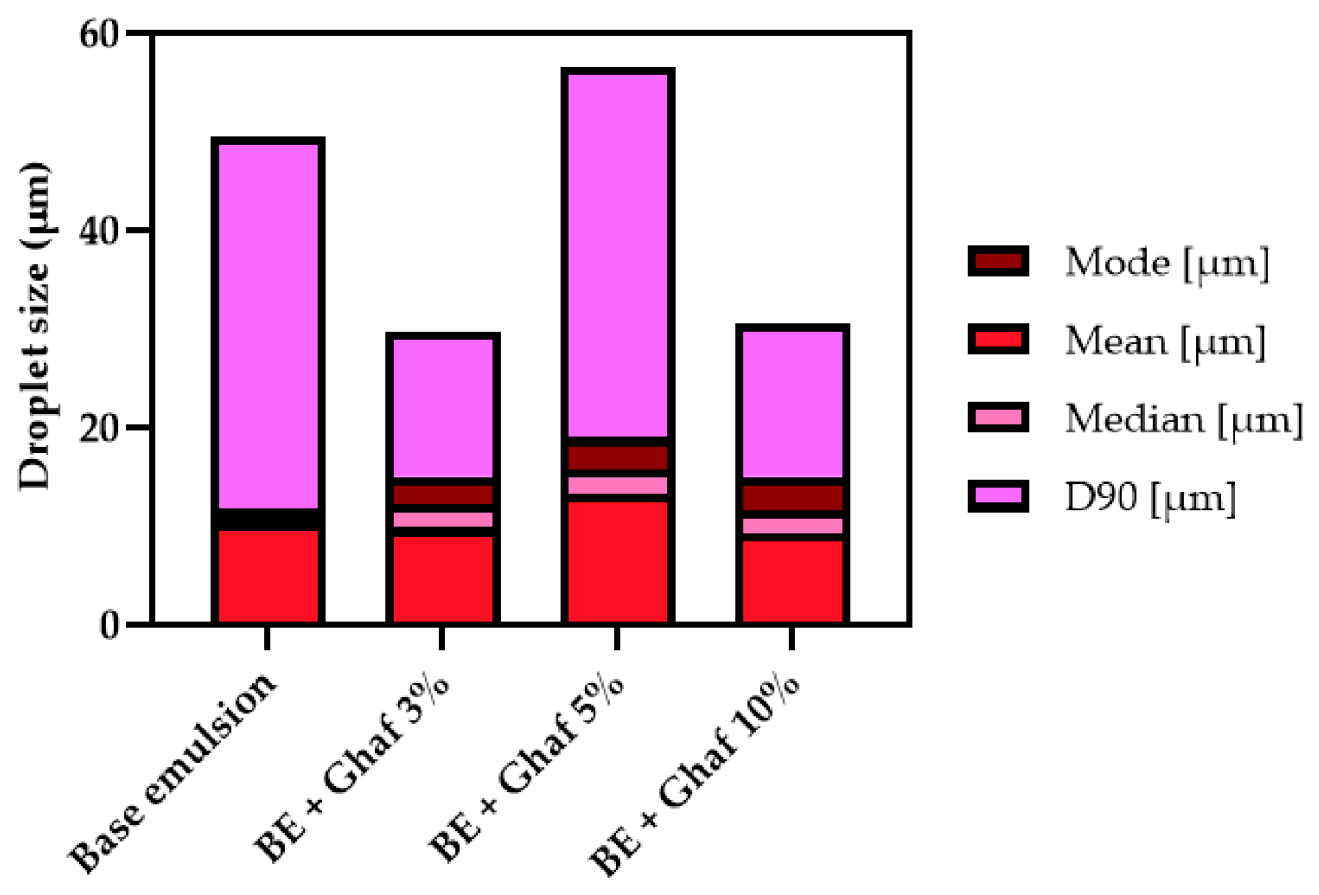
Droplet size distribution parameters of base emulsion (BE) and Ghaf-containing emulsions corresponding to the incorporation of a 2 mg/mL aqueous Ghaf extract solution at 3, 5, and 10% (*w*/*w*), yielding final extract concentrations of 60, 100, and 200 μg/mL, respectively. Median, mode, mean droplet diameter, and D90 values were determined by laser diffraction granulometry and expressed as the mean of three independent measurements.

The droplet size distribution of the base emulsion and of formulations containing the Ghaf extract solution at 3%, 5%, and 10% (*w*/*w*) was assessed by laser diffraction granulometry. As shown in Figure 9, the measured parameters indicate a consistent micrometric size distribution across all formulations, with median values ranging between 11.05 and 15.78 µm.

Although slight numerical variations were observed, especially an increase in the median and mode values in the emulsion containing Ghaf solution at 5% (*w*/*w*) formulation, the differences remain within the expected experimental variability and do not indicate a systematic or concentration-dependent shift in droplet size. Importantly, neither the mean diameter nor the variability among independent measurements exhibited a progressive trend with increasing extract concentration. The reported standard deviation values in Table 6 (remaining within the narrow interval 0.44–0.52 µm) refer to the variability between replicate measurements, highlighting the high reproducibility of the laser diffraction analysis across all samples. This observation suggests that the particulate stabilization mechanism provided by VIVAPUR^®^ CS TEX EASY controls droplet formation and stabilization and is not disrupted by extract loading.

From a formulation perspective, this structural stability, together with the consistent rheological behavior and physical stability described above, is a desirable feature for consistent texture, stability, and sensorial performance in cosmetic applications.

## 4. Discussion

This study identifies Ghaf extract as a source of bioactive compounds characterized by a promising in vitro antioxidant profile and enzyme-modulating properties relevant to skin aging. By investigating these specific activities, the present work provides a preliminary scientific framework that supports the potential of Ghaf as a naturally derived active for anti-aging dermocosmetics.

The strong antioxidant activity demonstrated by the Ghaf extract in the DPPH assay and further confirmed by the complementary ABTS and ORAC assays was consistent with its elevated total phenolic content determined by the Folin–Ciocalteu assay. Oxidative stress is widely recognized as one of the principal drivers of both intrinsic and photoinduced skin aging through the excessive production of reactive oxygen species (ROS), which promote lipid peroxidation, protein oxidation, DNA damage and activation of signaling pathways leading to extracellular matrix degradation [71]. Under identical experimental conditions, Ghaf extract exhibited antioxidant activity exceeding that of the reference antioxidant, suggesting the coexistence of multiple complementary antioxidant constituents acting synergistically rather than the presence of a single highly active compound. Similarly, the inhibitory activity observed against elastase, collagenase, hyaluronidase and tyrosinase can be related to the phytochemical profile identified by UHPLC–DAD–HRMS/MS analysis. Rather than attributing these biological activities generically to the overall polyphenolic fraction, the mechanistic interpretation was based primarily on metabolites identified with high confidence (MSI Levels 1 and 2), whereas metabolites assigned only at the compound-class level (MSI Level 3) were not considered for compound-specific biological interpretation. These confidently identified metabolites provide plausible mechanistic explanations for the observed activity of the extract. Among the compounds, catechins such as the unequivocally identified epicatechin (EC), and the putatively annotated epicatechin gallate (ECG) and epigallocatechin gallate (EGCG), are recognized among the most effective natural antioxidants [71,72]. Their radical-scavenging capacity depends on the abundance and spatial arrangement of hydroxyl groups within the flavan-3-ol structure, particularly the catechol and pyrogallol moieties, while the presence of a galloyl group at the C3 position markedly enhances hydrogen atom donation and electron transfer, making ECG and EGCG more potent antioxidants than non-galloylated catechins [73]. In addition, these catechins have been reported to inhibit tyrosinase through competitive interactions with the catalytic site and to reduce collagenase and elastase activity, thereby contributing to the preservation of extracellular matrix integrity [74].

Phenolic acids unequivocally identified in the Ghaf extract may also contribute to the biological profile. Gallic acid, owing to its trihydroxylated aromatic structure, exhibits remarkable radical-scavenging properties [75] and has been reported to inhibit collagenase, elastase [76] and tyrosinase [77]. Its inhibitory activity has been attributed to extensive hydrogen bonding with amino acid residues within enzyme catalytic pockets, while the free carboxyl group has also been proposed to coordinate the catalytic zinc ion of collagenase, thereby interfering with enzyme activity [78]. Likewise, the putatively annotated *p*-coumaric acid represents one of the best-characterized naturally occurring tyrosinase inhibitors. Because of its structural similarity to *L*-tyrosine, it competes with the physiological substrate for enzyme binding [79] while simultaneously acting as an efficient hydrogen donor during free-radical scavenging [80].

Several flavonoid glycosides putatively annotated in the Ghaf extract may further explain the activities observed. Myricetin glycosides, including myricetin-3-*O*-β-galactopyranoside and myricitrin, possess strong antioxidant properties resulting from both radical scavenging and transition-metal chelation [81,82]. Beyond these direct antioxidant effects, myricetin derivatives have been shown to inhibit the phosphorylation of p38 and JNK mitogen-activated protein kinases (MAPKs), thereby suppressing AP-1 activation through reduced c-Fos and c-Jun expression and ultimately decreasing MMP-1 production, one of the major enzymes responsible for collagen degradation during skin aging [83]. Similarly, rutin (MSI Level 1) and quercetin (MSI Level 2) derivatives possess ortho-dihydroxyl groups within the B-ring that efficiently chelate Fe^2+^ and Cu^2+^ ions, limiting metal-catalyzed ROS formation [84,85]. Molecular docking studies have additionally demonstrated that rutin can directly interact with elastase, hyaluronidase and tyrosinase, supporting its multifunctional anti-aging potential [85]. Other flavonoids putatively annotated in the extract, including patulitrin and apigenin-6-*C*-glucoside, have also been associated with anti-melanogenic and extracellular matrix-preserving activities. In particular, patulitrin has been shown to chelate the copper ions located within the tyrosinase active site, preventing substrate oxidation [86], whereas apigenin derivatives have demonstrated inhibitory effects against collagenase, elastase and hyaluronidase [87].

Collectively, these observations suggest that the broad antioxidant activity together with the inhibition of collagenase, elastase, hyaluronidase and tyrosinase likely results from the simultaneous action of these complementary mechanisms rather than from a single bioactive constituent. This presence of potentially effective compounds on multiple targets involved in the skin aging process is an advantage of botanical extracts in dermocosmetic applications, where oxidative stress, extracellular matrix degradation and dysregulated melanogenesis are closely interconnected biological processes.

The present findings are also consistent with previous investigations on polyphenol-rich botanical extracts incorporated into cosmetic formulations [88,89,90]. For example, creams containing *Vitis vinifera* grape stem extract, characterized by a phytochemical profile rich in catechins, gallic acid, rutin and quercetin derivatives comparable to that identified in Ghaf extract, retained substantial antioxidant and anti-aging activity following formulation. Although antioxidant capacity was partially reduced compared with the pure extract, likely because of the limited solubility or reduced stability of certain phenolic constituents within the emulsion, elastase inhibition remained largely preserved and tyrosinase inhibition was maintained at higher extract concentrations [91]. These observations suggest that polyphenol-rich phytocomplexes can preserve biological activity after incorporation into semisolid formulations and support the hypothesis that Ghaf extract may exhibit comparable functionality following incorporation into topical dermocosmetic products.

Based on these encouraging findings, a preliminary formulation study was intentionally designed to assess whether adding the extract to the selected cosmetic vehicle affected the formulation’s physicochemical stability, rheological behavior, and organoleptic characteristics. The results demonstrated the technological feasibility of incorporating the extract into a sustainable cosmetic emulsion, justifying further investigation of Ghaf extract in this field. Although these experiments were not designed to evaluate the biological performance of the finished formulation, they represent an essential preliminary step toward future formulation-oriented investigations. Moreover, its botanical origin, sustainable availability and traditional ethnopharmacological use further increase its attractiveness within the growing market for naturally derived dermocosmetic ingredients [92].

Nevertheless, several limitations should be acknowledged. First, as with all plant-derived materials, the phytochemical composition of Ghaf extract may be influenced by seasonal and environmental factors. Although experimental variability was minimized in the present study by standardized harvesting conditions, controlled tissue proportions and a reproducible extraction procedure, future industrial development will require implementation of standardized raw-material specifications together with phytochemical quality-control strategies, including chromatographic fingerprinting and/or quantitative determination of selected marker compounds, to ensure long-term batch-to-batch consistency.

Furthermore, the present study was intentionally conceived as a proof-of-concept evaluation of Ghaf as a potential dermocosmetic active aimed at providing the first comprehensive biochemical screening of Ghaf extract together with a preliminary formulation study. To this end, the experimental design was intentionally focused on mapping the primary upstream processes of skin aging (oxidative stress, extracellular matrix remodeling, and hyperpigmentation) through robust cell-free assays. Building upon these promising baseline data, the subsequent assessment of anti-aging efficacy in more physiologically relevant cellular models, together with formulation stability, release kinetics, and skin permeation studies, represents a valuable and logical direction for future research. These next steps will build on the solid premises established herein to achieve a more comprehensive characterization of the Ghaf extract and its finished dermocosmetic applications.

## 5. Conclusions

The present study demonstrates that Ghaf extract exhibits promising in vitro antioxidant and enzyme-modulating properties relevant to skin aging, supported by a phytochemical profile rich in bioactive polyphenolic compounds. In combination with the successful incorporation of the extract into a stable and sustainable Pickering emulsion, these findings provide a solid scientific rationale for the development of Ghaf as a botanical ingredient for dermocosmetic applications. These promising outcomes pave the way for future translational developments. Moving forward, the characterization of Ghaf-based dermocosmetics will logically benefit from standardization of the botanical raw material to ensure batch-to-batch reproducibility as well as advanced formulation studies investigating long-term chemical stability, release kinetics and skin permeation. Collectively, the present findings successfully establish an important proof of concept and provide a solid foundation for the design of sustainable Ghaf-based dermocosmetic formulations.

## Figures and Tables

**Table 1:** Percentage and weight of final Ghaf unified powder.

Plant Portion	Total Weight (g) of Obtained Powders	Percentage of Each Portion in Total (%)	Weight of Each Portion (g) Used
Twigs	3.77	8.2	0.082
Roots	7.43	16.2	0.162
Leaves	32.01	70.0	0.700
Bark	2.53	5.5	0.055
Total	45.74	100	1

**Table 2:** Base emulsion percentage composition (% *w*/*w*).

Ingredient	Phase	Quantity
Demineralized water	Aqueous	85.52
Glycerin	Aqueous	5.00
Potassium sorbate	Aqueous	0.20
VIVAPUR^®^ CS TEX EASY	Aqueous	5.50
Sunflower oil	Oil	2.53
Plum oil	Oil	1.26

**Table 3:** Base emulsions percentage composition (% *w*/*w*) containing Ghaf extract as aqueous solution (2 mg/mL) at 3, 5, and 10% (*w*/*w*), yielding final extract concentrations of 60, 100, and 200 μg/mL, respectively.

Ingredient	Phase	BE + Ghaf 3%	BE + Ghaf 5%	BE + Ghaf 10%
Demineralized water	Aqueous	82.52	80.52	75.52
Glycerin	Aqueous	5.00	5.00	5.00
Potassium sorbate	Aqueous	0.20	0.20	0.20
VIVAPUR^®^ CS TEX EASY	Aqueous	5.50	5.50	5.50
Ghaf solution 2 mg/mL	Aqueous	3.00	5.00	10.00
Sunflower oil	Oil	2.53	2.53	2.53
Plum oil	Oil	1.26	1.26	1.26

BE: base emulsion.

**Table 4:** HPLC-HRMS data of compounds detected in Ghaf extract.

N°	RT (min)	[M-H]^−^	Formula	Error (ppm)	Diagnostic Products Ion (m/z)	Name	Class	IL	Ref.
1	0.56	209.0305	C_6_H_10_O_8_	1.40	115.0039, 89.0246, 85.0297, 71.0140	Galactaric acid ^b^	Dicarboxylic acid	4	[38]
2	0.85	191.0216	C_6_H_8_O_7_	2.24	111.0089, 87.0089, 85.0296	Citric acid	Tricarboxylic acids	2	[39]
3	1.33	169.0145	C_7_H_6_O_5_	1.47	125.0247, 97.0297, 81.0343, 79.0192, 69.0347	Gallic acid	Phenolic acids and derivatives	1	std *
4	2.82	305.0650	C_15_H_14_O_7_	−3.6	179.0334, 167.0376, 139.0372, 137.0254, 125.0244, 109.0281	(Epi)gallocatechin	Catechins	2	[40]
5	6.12	451.2191	-	-	405.2136, 389.1100, 287.0162, 179.0567, 161.0463	Unknown	-	4	-
6	7.67	325.0936	C_15_H_18_O_8_	2.49	163.0405, 119.0505	Cumaric acid O-glucoside	Hydroxycinnamic acid derivative	2	[41]
7	8.33	289.0724	C_15_H_14_O_6_	2.16	245.0825, 221.0826, 205.0511, 203.0719, 161.0611, 151.0389, 125.0245, 123.0454, 109.0297, 97.0296	(Epi)catechin	Catechins	1	std *
8	8.72	457.0773	C_22_H_18_O_11_	0.44	305.0654, 169.0128, 125.0230	(Epi)gallocatechin gallate	Catechins	2	[42]
9	8.90	431.1928 ^a^	C_19_H_30_O_8_	1.92	385.1875, 223.1343, 205.1241, 153.0926	Drovomifoliol-O-glucoside (roseoside)	Sesquiterpenoid glycoside	2	[41]
10	9.01	197.0459	C_9_H_10_O_5_	2.70	169.0148, 125.0247, 124.0169	Ethyl gallate	Ellagitannins	2	[43]
11	9.21	557.2253	C_26_H_38_O_13_	3.23	-	Unknown	-	4	-
12	9.41	163.0405	C_9_H_8_O_3_	3.18	119.0505	p-Coumaric acid	Hydroxycinnamic acids	2	[44]
13	10.50	479.0841	C_21_H_20_O_13_	1.38	316.0230, 271.0252	Myricetin 3-O-beta-D-galactopyranoside	Flavonoid-glycosides	2	[45]
14	11.26	463.0891	C_21_H_20_O_12_	1.71	316.0232, 271.0256, 179.0565	Myricitrin	Flavonoid-glycosides	2	[46]
15	11.65	577.1574	C_27_H_30_O_14_	2.77	457.1149, 413.0885, 293.0463	2″-O-rhamnosylvitexin	Flavonoid-glycosides	2	[42]
16	11.68	609.1479	C_27_H_30_O_16_	2.72	300.0282	Rutin	Flavonoid-glycosides	1	std *
17	11.80	463.0889	C_21_H_20_O_12_	1.65	301.0335, 300.0256	Quercetin-3-*O*-galactoside (hyperoside)	Flavonoid-glycosides	2	[45]
18	12.15	493.0998	C_22_H_22_O_13_	2.15	330.0389, 315.0155	Patuletin-7-O-glucoside	Flavonoid-glycosides	2	[45]
19	12.53	567.2099	C_27_H_36_O_13_	3.52	457.1146, 383,0775, 353,0668, 341.0668, 293.0461	Dimethoxylflavanone derivate	Flavonoid derivates	3	[47]
20	13.31	415.1979	-	-	255.4785, 240.5105, 137.0976	Unknown	-	4	-
21	13.61	547.1471	C_26_H_28_O_13_	2.64	383.0777	Unknown	-	4	-
22	13.97	577.1577	C_27_H_30_O_14_	-	559.1448, 457.1127, 395.0760, 293.0451, 269.0450	Apigenin-di-C-glucoside isomer	Flavonoid-glycosides	2	[48,49]
23	14.51	577.1578	C_27_H_30_O_14_	-	559.1445, 457.1126, 395.0762, 293.0457, 269.0444	Apigenin-di-C-glucoside isomer	Flavonoid-glycosides	2	[48,49]

* std: standard compound; ^a^ [M + HCOOH − H]^−^; ^b^ annotations based on accurate mass and molecular formula only (no MS/MS) MSI level 4.

**Table 5:** Physical study of base emulsion (BE) and emulsion with Ghaf extract at different percentages for one week. Ghaf-containing emulsions correspond to the incorporation of a 2 mg/mL aqueous Ghaf extract solution at 3, 5, and 10% (*w*/*w*), yielding final extract concentrations of 60, 100, and 200 μg/mL, respectively.

Duration	7 Days						
	Storage Condition						
Parameter	4 °C	25 °C	45 °C	Parameter	4 °C	25 °C	45 °C
Appearance				Odor			
Base emulsion	Semisolid	Semisolid	Semisolid	Base emulsion	Plum oil scent	Plum oil scent	Plum oil scent
BE + Ghaf 3%	Semisolid	Semisolid	Semisolid	BE + Ghaf 3%	Plum oil scent, mild herbal	Plum oil scent, mild herbal	Plum oil scent, mild herbal
BE + Ghaf 5%	Semisolid	Semisolid	Semisolid	BE + Ghaf 5%	Plum oil scent, strong herbal	Plum oil scent, strong herbal	Plum oil scent, strong herbal
BE + Ghaf 10%	Semisolid	Semisolid	Semisolid	BE + Ghaf 10%	Plum oil scent, strong herbal	Plum oil scent, strong herbal	Plum oil scent, strong herbal
Color				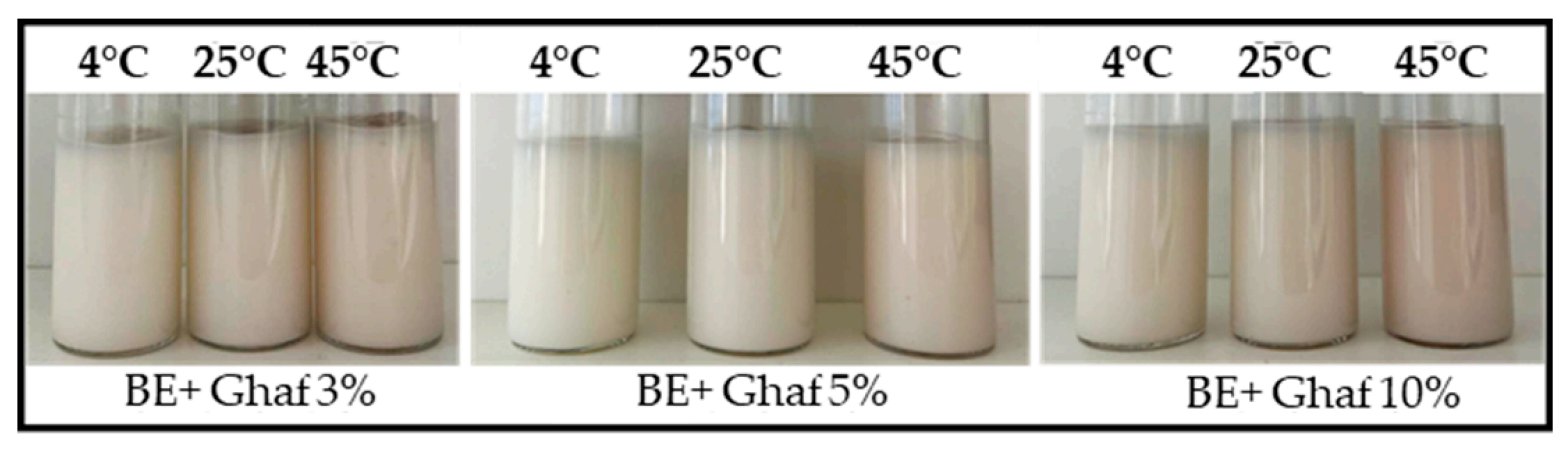			
Base emulsion	Off-white	Off-white	Off-white				
BE + Ghaf 3%	Off-white	Off-white	Off-white				
BE + Ghaf 5%	Off-white	Off-white	Off-white				
BE + Ghaf 10%	Off-white	Light greyish	Light beige				

**Table 6:** Droplet size means ± standard deviation of three measurements of base emulsion (BE) and Ghaf-containing emulsions corresponding to the incorporation of a 2 mg/mL aqueous Ghaf extract solution at 3, 5, and 10% (*w*/*w*), yielding final extract concentrations of 60, 100, and 200 μg/mL, respectively.

	Mean (µm)	Std. Dev.
Base emulsion	10.43	0.50
BE + Ghaf 3%	9.81	0.44
BE + Ghaf 5%	13.24	0.51
BE + Ghaf 10%	9.29	0.46

BE: base emulsion.

## Data Availability

The authors declare that the data supporting the findings of this study are available within the paper. Should any raw data files be needed in another format they are available from the corresponding author upon reasonable request.
